# Glial biomarkers improve classification of cognitive impairment: an explainable artificial intelligence study using CSF biomarkers

**DOI:** 10.3389/fneur.2026.1787915

**Published:** 2026-04-23

**Authors:** Merve Türkegün Şengül, Dilara Nemutlu Samur

**Affiliations:** 1 Department of Biostatistics and Medical Informatics, Faculty of Medicine, Alanya Alaaddin Keykubat University, Antalya, Türkiye; 2 Department of Medical Pharmacology, Faculty of Medicine, Alanya Alaaddin Keykubat University, Antalya, Türkiye

**Keywords:** Alzheimer’s disease, cognitive impairment, explainable artificial intelligence, glial biomarkers, machine learning, neuroinflammation

## Abstract

**Background:**

Alzheimer’s disease (AD) is increasingly recognized as a disorder involving not only amyloid and tau pathology but also glial activation and neuroinflammation. Biomarkers reflecting these processes may improve the classification of clinical cognitive impairment. This study evaluated the diagnostic value of glial biomarkers, alongside cerebrospinal fluid (CSF) biomarkers, for distinguishing cognitively normal individuals from those with clinical dementia rating scale (CDR)-defined very mild or mild dementia.

**Methods:**

Data from 333 adults aged ≥60 years were obtained from the Knight Alzheimer’s Disease Research Center’s longitudinal, open-access dataset. Seven multimodal models integrating CSF biomarkers, glial biomarkers, and clinical features were developed using machine-learning approaches. A hybrid model incorporating feature selection was applied, and model interpretability was assessed. Classification performance was evaluated using AUC, accuracy, recall, precision, and F1-score.

**Results:**

Incorporating all biomarkers, the model achieved the highest performance (AUC = 0.959; accuracy = 0.912), followed by a parsimonious hybrid model (clustering, cystatin C, age, tau, Aβ42, sex) with comparable performance (AUC = 0.951; accuracy = 0.868; *p* = 0.309). According to SHAP analysis, tau and cystatin C were the most influential features in both models for clinical impairment classification.

**Conclusion:**

Glial biomarkers significantly enhance diagnostic classification of CDR-defined clinical cognitive impairment beyond core CSF biomarkers. Parsimonious and interpretable machine-learning models achieve performance comparable to more complex approaches, supporting their potential use in clinical stratification frameworks.

## 1 Introduction

Cognitive impairment (CI) in older adults represents a major clinical and public health challenge ranging from mild cognitive impairment (MCI) and dementia (1). MCI is considered to be a transitional state between normal aging and dementia, especially Alzheimer’s disease (AD) (2). It is associated with an elevated risk of progression to AD dementia, particularly when AD-related biomarkers are present (3). Accurate identification and stratification of individuals along this cognitive spectrum therefore remain major challenges in clinical research. Among the neurodegenerative disorders underlying progressive cognitive decline, AD is the most common cause and extensively studied etiology (4). AD is a progressive neurodegenerative disorder characterized by amyloid-β42 (Aβ42) deposition, tau hyperphosphorylation, synaptic dysfunction, and neuronal loss (5). Despite ongoing scientific advances, a definitive diagnosis of AD can be confirmed only post-mortem by histopathological verification of amyloid plaques and neurofibrillary tangles, whereas clinical diagnosis in the early stages is unreliable owing to subtle, nonspecific, and heterogeneous symptoms (6, 7). Early and accurate detection is critical because the pathological cascade begins 10–20 years before symptom onset, a period during which neurodegeneration is still limited, and therapeutic interventions are more likely to be effective (8). Accordingly, sensitive biomarkers that capture these preclinical changes are central to advancing AD-related research and disease-modifying strategies, shifting the focus toward identifying targets at the earliest stages of the disease (9).

Due to its direct contact with the brain’s extracellular environment, cerebrospinal fluid (CSF) serves as one of the most accessible and informative fluid reflecting molecular changes in the central nervous system (10). Thus far, three CSF biomarkers, Aβ42, total-tau (tau), and phosphorylated-tau (p-tau), have been found to have the highest diagnostic potential (11). However, increasing evidence suggests that AD is not solely a neuronal proteinopathy but also a disorder of chronic glial activation, neuroinflammation, and disrupted neuron–glia crosstalk, which precede obvious neurodegeneration and may be detectable years before clinical onset (12).

Increasing evidence has highlighted the critical role played by non-neuronal cells, particularly glial cells, in the neurodegenerative process. Traditionally considered supportive cells, microglia and astrocytes are now considered to be active regulators of brain homeostasis, immunity, and synaptic support (13). In AD, these cells undergo marked phenotypic transitions: microglia shift toward pro-inflammatory states, releasing cytokines and chemokines that exacerbate neuronal injury, while astrocytes become reactive, lose homeostatic functions, and amplify neuroinflammation. Their reciprocal activation generates a self-sustaining inflammatory loop that accelerates synaptic dysfunction and neuronal loss (14). In parallel, the classical AT(N) framework (A for amyloid pathology, T for tau pathology, and N for neurodegeneration) has recently been expanded to incorporate neuroinflammation, giving rise to the AT(N)I classification. The added “I” component captures inflammatory and glial activation biomarkers, enabling neuroinflammation to be monitored as a fourth biological axis of AD (15). This shift reflects growing recognition that glial responses are not downstream consequences but early and mechanistically relevant drivers of AD pathogenesis. Accordingly, several glial-related CSF proteins, such as glial fibrillary acidic protein (GFAP), chitinase-3-like-1 protein (YKL-40), S100 calcium binding protein B (S100B), and soluble triggering receptor expressed on myeloid cells 2 (sTREM2), have emerged as candidate biomarkers reflecting neuroinflammation and glial activation states (16, 17).

Cognitive status is typically staged using the Clinical Dementia Rating (CDR) scale, which classifies individuals from cognitively unimpaired (CDR 0) to very mild or mild impairment (CDR 0.5–1) in AD research (18). Determining whether glial activation biomarkers improve discrimination across these CDR-defined clinical stages may clarify their translational relevance beyond core amyloid and tau biomarkers.

Machine learning (ML) algorithms are one of the artificial intelligences (AI) techniques used to select the models that best fit a set of observations. The use of ML methods such as support vector machines, random forest, and artificial neural networks has rapidly increased in the classification of AD neuroimaging data and the prediction of MCI to support clinical classification in cognitive disorders (19, 20). However, these methods have certain limitations. ML inherently relies on data; this can lead to imbalances in the class distribution of the outcome variable. Models trained on such imbalanced datasets tend to overestimate the majority class while leading to lower sensitivity and higher false positive rates for the minority class. Eliminating this bias is critically important. On the other hand, more complex ML models such as deep learning have high predictive power, but they are difficult to adopt in clinical practice because they generally operate as “black boxes” (21). Combining the eXtreme Gradient Boosting (XGBoost) algorithm, one of the ML methods that addresses the class imbalance problem encountered in clinical applications and reduces diagnostic performance at the algorithmic level, together with the Shapley additive explanations (SHAP) method, which eliminates the black box problem, makes the decision mechanisms of models transparent and helps identify effective features (22). This enables the generation of easy-to-understand predictions for healthcare professionals.

AI and ML approaches have been successfully applied in numerous biomarker discoveries related to dementia. These methods shed light on the reliable analysis of complex, multimodal data sets and the identification of new patterns or potential biomarkers (23). Studies show that AI and ML algorithms, when combined with new biomarkers that go beyond traditional Aβ42 and tau proteins, have the potential to revolutionize AD diagnosis (24). Glial biomarkers may not only correlate with cognitive decline but may also provide independent diagnostic value by capturing inflammatory states that are not reflected by amyloid or tau biomarkers. Accordingly, there is growing interest in determining whether glia-derived biomarkers can enhance diagnostic performance either by complementing or potentially surpassing traditional core CSF biomarkers. Integrating glial biomarkers with classical AD biomarkers may therefore enable the development of multidimensional biomarker models that better represent the distinct biological axes of the disease. However, the comparative diagnostic performance of glial versus traditional biomarkers remains insufficiently defined, and it is unclear whether glial biomarkers improve disease classification when incorporated into machine-learning-based multivariate models alongside core biomarkers and demographic variables such as age and sex.

To address these gaps, we aimed to investigate whether glial biomarkers provide additional value to traditional CSF biomarkers in distinguishing cognitively unimpaired individuals from those with clinical cognitive impairment using the XGBoost-SHAP framework, which is considered superior to other machine learning methods. Rather than modeling biomarker-confirmed AT(N)-defined AD pathology, our objective is to determine whether glial activation biomarkers enhance machine-learning-based classification of clinically staged cognitive status within an research cohort. In this context, we sought to answer the following questions using XGBoost: (i) Does the combination of glial activation and traditional CSF biomarkers change the classification performance for CI? (ii)How successful are glial biomarkers alone in classification performance for CI? (iii)What is the classification performance of traditional core biomarkers alone? (iv) What are the contributions of age and sex to CI classification performance when age and sex are used in combined with glial and traditional core biomarkers?

This work aims to clarify the translational potential of glial activation biomarkers in characterizing clinical progression and provides an interpretable machine-learning framework for multidimensional biomarker integration in cognitive disorders.

## 2 Materials and methods

We implemented a robust machine learning framework that leverages the XGBoost algorithm to classify CDR-defined clinical CI using R v.4.2.1. It involves several key stages, including data splitting, data preparation, feature engineering, model training with hyperparameter optimization, evaluation, and interpretability analysis.

### 2.1 Participants

We used the open-access dataset called “AlzheimerDisease” from the longitudinal clinical study conducted by Craig-Schapiro et al. at the Knight Alzheimer’s Disease Research Center at Washington University (WU-ADRC) in the study (25). The primary objective of the original study was to determine and discrimination power of CSF and other biomarkers various combinations for the diagnosis and prognosis of AD. The data used in this work are accessible via the R package (CRAN) “AppliedPredictiveModeling” and represent a curated, modified version of the original study values specifically prepared for predictive modeling (26). This version includes a subset of 333 subjects with complete demographic and laboratory results, categorized by clinical cognitive status. By using this standardized and publicly available version, we ensure the reproducibility of our machine-learning framework within a well-characterized research cohort.

The original study included individuals aged ≥60 years who were in satisfactory general health, who could contribute significantly to dementia research, and who did not have other neurological, psychiatric, or major medical diagnoses. Clinical diagnosis was evaluated based on criteria from the National Institute of Neurological and Communicative Diseases and Stroke-Alzheimer’s Disease and Related Disorders Association (NINCDS-ADRDA) (27). Cognitive status was assessed using the Clinical Dementia Rating (CDR). If the CDR is 0, it indicates no dementia. These participants were neuropsychologically normal, functionally independent, and clinically healthy, constituting the control group (*N* = 242). If CDR is 0.5, it evaluates very mild dementia, and if CDR is 1, it evaluates mild dementia (28). In the modified version of the dataset distributed through the *“AppliedPredictiveModeling: AlzheimerDisease,”* individuals with CDR 0.5 (very mild dementia) and CDR 1 (mild dementia) are combined into a single “Impaired” category (*N* = 91), and the original CDR subgroup labels are not available.

### 2.2 Biomarkers and models

Craig-Schapiro’s study included traditional CSF analytes such as Aβ42, tau, and p-tau, as well as a total of 125 inflammatory, neuronal damage, and glial activation biomarkers, along with demographic characteristics such as age and sex (25). From this dataset, we created two biomarker panels with glial and core biomarkers to incorporate the glial cell-derived inflammatory response. The first is called a “core biomarker,” and it includes tau, p-tau, and Aβ42, reflecting the neuropathological basis of AD. The second is a “glial biomarker” panel representing the neuroinflammation-glial activation axis, including clusterin (ApoJ), S100B, calbindin, NrCAM, osteopontin, sortilin, and cystatin C. The dataset provides a comprehensive structure at both the clinical and biomarker levels, thereby forming an analytical foundation suitable for evaluating CDR-defined clinical CI classification using machine learning algorithms.

Seven models, each constructed from combinations of core CSF biomarkers, glial CSF biomarkers, and clinical features (age and sex), were systematically evaluated using XGBoost, an advanced machine learning algorithm, to identify the model with the highest classification performance. This combination enables testing, using comparative models, of the independent or complementary classification value of core pathological processes and glial and inflammatory mechanisms. These models were defined as follows:

Model 1: Core biomarkers + glial biomarkers + age+sex.

Model 2: Core biomarkers +age+sex.

Model 3: Glial biomarkers + age+sex.

Model 4: Core biomarkers + glial biomarkers.

Model 5: Core biomarkers.

Model 6: Glial biomarkers.

Model 7: Hybrid model (LASSO+ XGBoost for core biomarkers + glial biomarkers + age+sex).

### 2.3 Statistical methods

#### 2.3.1 Data partitioning and preprocessing

The categorical outcome variable “Diagnosis” was converted to a factor with levels “Control” and “Impaired,” and sex was converted to a factor with levels “Female” and “Male.” Since the original data were z-standardized for age, no transformations were applied to age or to other numeric variables. There were no observations missing in the original data.

The dataset was divided into training (80%) and testing (20%) sets. A stratified sampling technique based on the outcome variable was used to preserve the original prevalence of patients in the training and test sets. After the stratified 80/20 split, the independent test set consisted of 49 control and 19 impaired individuals. This test set was used for performance evaluation of all models.

#### 2.3.2 Machine learning algorithm: XGBoost

We implemented a supervised ML framework based on XGBoost to distinguish cognitively impaired participants from controls. XGBoost combines multiple weak models (small decision trees) sequentially to create a powerful prediction model based on the principle of gradient boosting. In this approach, trees are added to the model one by one; each new tree improves the model iteratively by correcting the mistakes made by previous trees. The process ends when the specified number of trees is reached or when performance improvement stops Also, XGBoost is known to provide an optimal balance between accuracy and training speed compared with other gradient boosting algorithms and is widely used in biomarker-based models where classification accuracy is important. Therefore, seven XGBoost models have been established with all biomarker sets (22).

#### 2.3.3 Handling class imbalance via cost-sensitive learning

Without resorting to resampling methods that carry a risk of data leakage, XGBoost hyperparameter optimization can assign higher weights to the minority class. This reduces the false positive rate and yields more accurate classifications. In the training set, the class-weight coefficient (2.68) was calculated based on the counts of control and impaired cases to reduce class imbalance. This weight was used in all XGBoost models.

#### 2.3.4 Hyperparameter optimization and cross-validation

The same XGBoost specification was used in all models. Various measures were implemented to mitigate overfitting. The tree-based structure was kept simple to limit the risk of overfitting. Additionally, L2 (lambda = 5) and L1 (alpha = 2) penalties were defined in the XGBoost engine to further regularize the model. This minimized the formation of overly complex tree structures and excessive adaptation to noisy variables, particularly in small-sample clinical data.

The hyperparameter space was scanned using a grid consisting of 30 points generated by Latin hypercube sampling. The grid search was conducted for each model utilizing 5-fold stratified cross-validation on the training data. The primary performance metric used was the Area Under the ROC Curve (AUC), and the final hyperparameter selection was based on the combination that yielded the highest cross-validation AUC. The XGBoost specification also incorporated an early stopping mechanism; that is, training was terminated prematurely if the validation performance did not improve over a specified number of iterations.

All feature selection and hyperparameter tuning processes were carried out solely within the training data using cross-validation to guarantee a distinct division between model creation and evaluation. The final models were subsequently evaluated on a held-out independent test set that was not used at any stage of model development. Nonparametric bootstrap resampling (2,000 iterations) of the test-set predictions was used to obtain 95% CIs for all performance metrics in order to measure uncertainty in performance estimations. Additional analyses assessing model stability, including learning-curve evaluation, are described in Section 2.3.8.

#### 2.3.5 Two-stage hybrid feature selection via LASSO

Biomarker measurements were obtained from a previously published dataset in which z-standardization had already been applied; therefore, no additional transformation or standardization was performed in the present analysis.

For the hybrid model, we implemented a two-stage strategy to identify the most effective predictors. *Stage 1:* Penalized Logistic Regression: We utilized the Least Absolute Shrinkage and Selection Operator (LASSO), a penalized regression method, and evaluated it in the study both as an independent machine learning model and for variable selection to determine the most effective biomarkers for distinguishing cognitively impaired from cognitively unimpaired individuals (29).

To ensure clinical validity, age and sex were specified as unpenalized variables (penalty.factor = 0) in the LASSO model, based on clinicians’ recommendations, whereas glial and core biomarkers were penalized (penalty.factor = 1). 10-fold cross-validation was applied to the training set to prevent overfitting during model optimization. To prevent data leakage, variable selection with LASSO was performed only on the training set. Variable selection was performed using the *lambda.1se* criterion, which produced a simpler model. Instead of interaction terms, the strongest predictors were selected from among basic biomarkers and demographic variables. Thus, a “clinically simpler (parsimonious) yet powerful” feature set was obtained, which will be retrained with XGBoost.

*Stage 2 (Non-linear Modeling):* The subset of biomarkers selected by LASSO was used as input for the XGBoost classifier. Thus, a hybrid model was created. We conducted repeated subsampling analyses on the training data to further investigate the robustness of the LASSO-based feature selection beyond cross-validation folds. Specifically, 500 stratified subsamples were generated, each containing 80% of the training set while preserving the original outcome distribution. In each iteration, the LASSO model was refitted using the *lambda.1se* selection criterion. For each biomarker, the selection frequency was calculated as the proportion of iterations in which the variable was retained in the model. This procedure provides an empirical estimate of feature-selection stability in the presence of potentially correlated biomarker panels.

#### 2.3.6 Final model training and statistical evaluation

The optimal hyperparameters determined during cross-validation were used to train the final XGBoost models on the entire training set. Optimal probability thresholds for classification were determined using Youden’s J index derived from ROC analysis on the training set. When determining threshold-dependent performance metrics, these thresholds were fixed and then applied unchanged to the independent test set for the purpose to prevent information leaking. The optimal probability thresholds ranged between 0.161 and 0.193 across models.

Model performance was evaluated on the independent test set using area under the receiver operating characteristic curve (AUC), accuracy, sensitivity, specificity, positive predictive value (PPV) and F1-score. Pairwise comparisons between model AUCs were performed using DeLong’s test. 95% confidence intervals were estimated using nonparametric bootstrap resampling (2,000 iterations) of the independent test-set predictions for all these performance metrics. In all these evaluations, the impaired group was defined as the positive group.

Model calibration was evaluated on the independent test set using multiple complementary measures. The Brier score was calculated to assess overall probabilistic accuracy. We performed calibration analyses on the independent test set using Brier scores and 10-bin reliability plots to evaluate the probabilistic accuracy of our models. Calibration was further quantified by calculating the calibration slope and intercept (Calibration-in-the-large, CITL). While an ideal calibration is represented by a slope of 1 and an intercept of 0, these metrics allowed us to identify any systematic over- or under-estimation in our model’s probability scaling, ensuring a more transparent assessment of model reliability.

#### 2.3.7 Explainable artificial intelligence: SHAP

A significant disadvantage of machine learning models is that decision-making processes often remain a “Black Box.” In other words, in clinical studies it may not be possible to determine from models developed using ML which biomarker contributes to classification decisions, in which direction, and to what extent. However, this information is critical. Therefore, we used SHAP, an explainable artificial intelligence (XAI) method, to clinically interpret the results obtained from the prediction model. Using this method, the contribution of each feature to the prediction model is quantified by SHAP values. The feature that contributes most to the model has the highest SHAP value (16, 30, 31). Each colored point in the SHAP summary plots corresponds to an observation. The Y-axis represents the features included in the model; the X-axis shows the SHAP values for these features. Features are ranked in descending order of importance based on their average absolute SHAP value. When the SHAP value is positive, it indicates that the relevant feature contributes to increasing the probability of being classified as impaired, while a negative SHAP value indicates a risk-reducing (protective) effect. Colors indicate a feature’s observed value: yellow tones represent high values, and purple tones represent low values. Thus, both the value of the variable and the direction and magnitude of its contribution to the model prediction can be visually assessed (30). SHAP values were computed using the training-set data from the final tuned models. The independent test set was reserved exclusively for model performance evaluation and was not used for interpretability analyses. This method facilitates interpretation of model predictions in a clinical context and identify features that may contribute meaningfully to model discrimination.

#### 2.3.8 Learning-curve analysis and overfitting assessment

An AUC-based learning-curve analysis was performed to evaluate model stability and potential overfitting as a function of training sample size for machine-learning models.

Subsamples stratified by diagnosis status were drawn from the training set at sampling rates of 10, 20, 40, 60, 80, and 100%. The models were then retrained on these subsets using the final hyperparameters. Multiple repetitions were performed for each training rate. AUC values were calculated on both the corresponding training subset and the fixed independent test set. The mean AUC values and their corresponding 95% confidence intervals were then estimated across repeated subsampling iterations. The train-test performance gap (GAP) was defined as the difference between the mean training AUC and the corresponding test AUC (GAP = *AUC_train_–AUC_test_*) for the purpose of calculating the divergence between training and test performance. Smaller GAP values indicate consistent performance between training and test data, whereas larger positive gaps may suggest potential overfitting. As a practical reference, persistent GAP values exceedingly approximately 0.10 would be considered suggestive of substantial overfitting.

## 3 Results

### 3.1 Baseline characteristics of participants and biomarkers across CDR-defined groups

Exploratory analyses revealed that levels of tau and p-tau, classic biomarkers commonly used in AD diagnosis, were elevated in the impaired group, while Aβ42 levels were decreased (Adj-*p* < 0.05). Among glial biomarkers, only osteopontin and sortilin values were found to be higher in the impaired group (Adj-p < 0.05), while no significant difference was observed between the groups for other glial biomarkers The mean age of the impaired group (75.28 ± 7.02) was higher than that of the control group (71.60 ± 7.40; Adj-*p* < 0.001). Furthermore, a significant relationship was observed between gender and groups (*p* = 0.037). The proportion of women was higher in the control group (Table 1).

**Table 1:** Distribution of biomarkers across study groups.

Biomarkers	Control (*n* = 242)	Impaired (*n* = 91)	Adj *p-*value
Aβ42	0.24 ± 0.92	−0.65 ± 0.92	**<0.001**
tau	−0.27 ± 0.89	0.73 ± 0.91	**<0.001**
p-tau	−0.21 ± 0.90	0.57 ± 1.03	**<0.001**
Calbindin	−0.06 ± 0.93	0.16 ± 1.16	1.000
Clusterin Apo J	−0.09 ± 0.96	0.24 ± 1.07	0.128
Cystatin c	0.09 ± 0.99	−0.25 ± 0.98	0.061
NrCAM	0.01 ± 1.01	−0.03 ± 0.98	1.000
Osteopontin	−0.11 ± 0.97	0.28 ± 1.03	**0.024**
S100b	−0.09 ± 1.00	0.24 ± 0.97	0.087
Sortilin	−0.11 ± 0.99	0.30 ± 0.96	**0.006**
Demographics	Control (*n* = 242)	Impaired (*n* = 91)	
Age	71.6 ± 7.4	75.28 ± 7.02	**<0.001**
Sex			
*Female*	157 (64.90%)	47 (51.60%)	**0.037**
*Male*	85 (35.10%)	44 (48.40%)	

Adj p, Adjusted *p*-values are Bonferroni-corrected. Bold values indicate statistically significant results.

**Figure 1:**
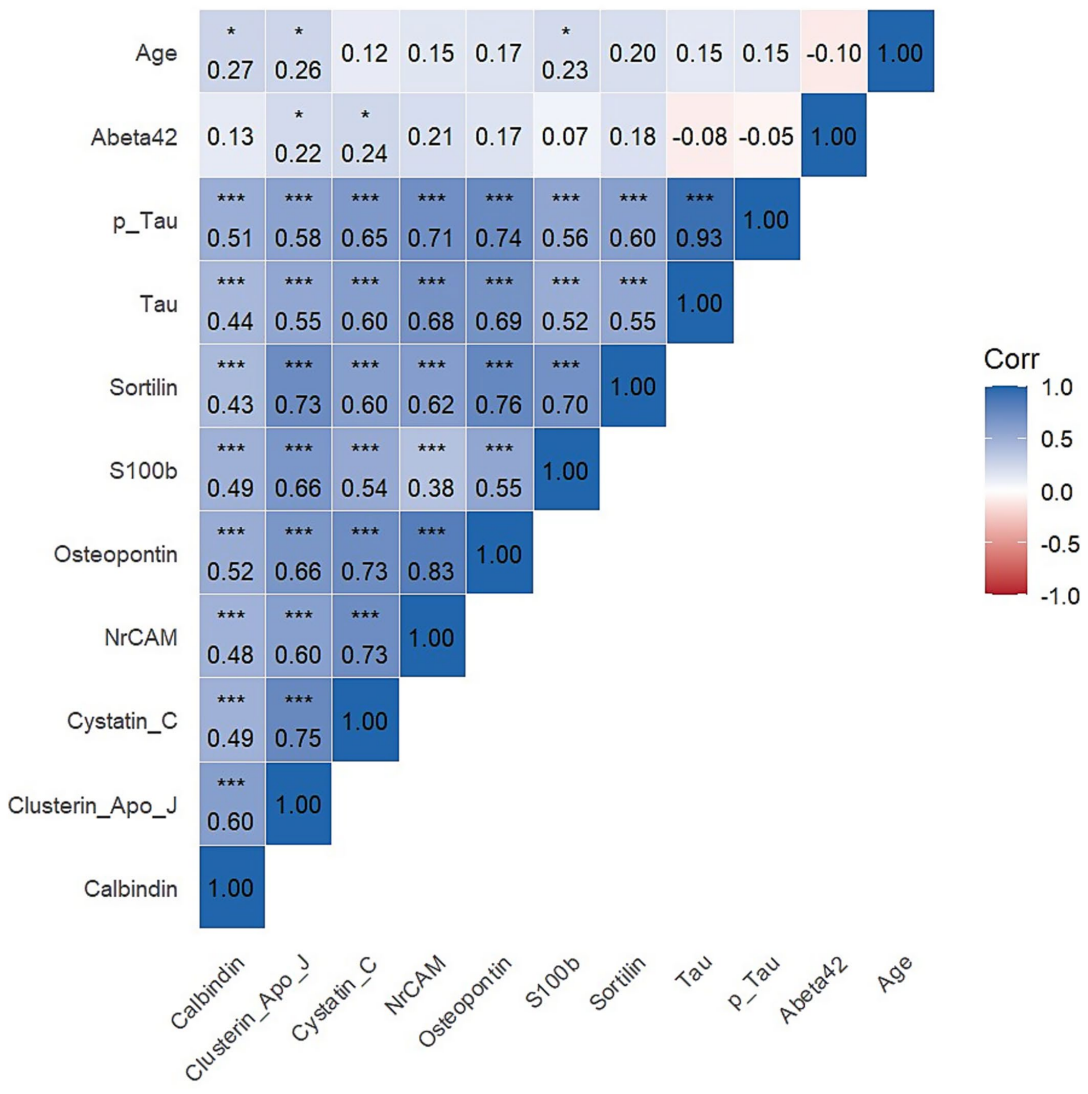
Heatmap for Pearson correlation coefficient.

The correlations between biomarkers in Figure 1 were evaluated. There is a very strong positive correlation between the traditional AD biomarkers tau and p-tau (*r* = 0.93). A very weak negative correlation was observed between Aβ42 and both tau and p-tau. It is noteworthy that all glial biomarkers are moderately and positively correlated with each other, and these correlations are statistically significant. The highest correlation was between osteopontin and NrCAM (*r* = 0.83, *p* < 0.05), while the lowest correlation was between S100b and NrCAM (*r* = 0.38, *p* < 0.05). Significant correlations were also found between traditional biomarkers and glial biomarkers. In particular, strong correlations were observed between osteopontin and p-tau (*r* = 0.74, *p* < 0.05) and between osteopontin and tau (*r* = 0.69, *p* < 0.05). Age showed statistically significant but weak correlations with the glial biomarkers calbindin (*r* = 0.27), clusterin apo J (*r* = 0.26), and S100b (*r* = 0.23).

### 3.2 Model performances

In this study, seven predictive models, each with different combinations of predictors, were developed to classify CDR-defined clinical CI. Six XGBoost models were fitted using core CSF biomarkers, glial biomarkers, and demographic variables, and a seventh “hybrid” model combined LASSO-based variable selection with XGBoost. The hybrid model, selected via LASSO, retained clusterin, cystatin C, age, tau, Aβ42, and sex as predictors associated with CDR-defined clinical CI.

As presented in Table 2, the discrimination results for Models 1, 4, and 7 were highly similar and could be considered comparable. Model 4, which included only core and glial biomarkers, achieved the highest AUC of 0.955 (95% CI: 0.902–0.990) and showed an accuracy of 89.7%, sensitivity of 84.2%, specificity of 91.8%, PPV of 80.0%, and an F1 score of 82.1%. Model 1, which included all biomarkers, age, and sex, produced nearly identical classification metrics to Model 4, with a slightly higher AUC of 0.959 (95% CI: 0.908–0.992).

**Table 2:** Predictive performance of the XGBoost models.

XGBoost Models	AUC (95% CI)	Accuracy (95% CI)	Sensitivity (95% CI)	Specificity (95% CI)	PPV (95% CI)	F1 score (95% CI)	Brier score (95% CI)
Model 1	0.959 (0.908–0.992)	0.912 (0.838–0.971)	0.895 (0.737–1.000)	0.918 (0.830–0.981)	0.810 (0.625–0.957)	0.850 (0.706–0.955)	0.109 (0.065–0.159)
Model 2	0.888 (0.800–0.955)	0.824 (0.721–0.912)	0.684 (0.467–0.895)	0.878 (0.778–0.962)	0.684 (0.450–0.889)	0.684 (0.476–0.837)	0.158 (0.102–0.217)
Model 3	0.830 (0.724–0.919)	0.691 (0.588–0.794)	0.684 (0.470–0.889)	0.694 (0.569–0.818)	0.464 (0.280–0.652)	0.553 (0.364–0.711)	0.176 (0.115–0.238)
Model 4	0.955 (0.902–0.990)	0.897 (0.824–0.971)	0.842 (0.667–1.000)	0.918 (0.830–0.981)	0.800 (0.609–0.955)	0.821 (0.667–0.933)	0.114 (0.068–0.165)
Model 5	0.908 (0.825–0.968)	0.838 (0.750–0.926)	0.737 (0.524–0.923)	0.878 (0.778–0.962)	0.700 (0.474–0.895)	0.718 (0.529–0.857)	0.146 (0.091–0.205)
Model 6	0.822 (0.703–0.925)	0.750 (0.647–0.853)	0.684 (0.462–0.895)	0.776 (0.654–0.889)	0.542 (0.333–0.739)	0.605 (0.412–0.760)	0.190 (0.125–0.256)
Model 7	0.951 (0.894–0.989)	0.868 (0.779–0.941)	0.842 (0.666–1.000)	0.878 (0.780–0.962)	0.727 (0.524–0.909)	0.780 (0.615–0.903)	0.114 (0.068–0.164)

Positive class is accepted as impaired group. CI: 95% confidence intervals with *n* = 2000 bootstrap resampling. Threshold-dependent metrics were computed at the ROC-optimized threshold, determined on the training set and applied to the independent test set.

Furthermore, Model 1, and 4 yielded the relatively lowest Brier scores (0.109 and 0.114, respectively). Model 7, a hybrid model combining LASSO-selected predictors with XGBoost, also demonstrated strong overall performance, with an accuracy of 86.8%, sensitivity of 84.20%, specificity of 87.8%, PPV of 72.7%, an F1-score of 78.0%, and an AUC of 0.951 (95% CI: 0.894–0.989). Moreover, the hybrid model demonstrated a relatively low Brier score (0.114) like Model 4. Model 2, which included core biomarkers, age, and sex, showed an accuracy of 82.4%, sensitivity of 68.4%, specificity of 87.8%, PPV of 68.4%, an F1-score of 68.4%, and an AUC of 0.888 (95% CI: 0.800–0.955). Model 3, which included glial biomarkers, age, and sex, demonstrated moderate overall performance, with an accuracy of 69.1%, sensitivity of 68.4%, specificity of 69.4%, PPV of 46.4%, an F1-score of 55.3%, and an AUC of 0.83.0 (95% CI: 0.724–0.919) and a Brier score of 0.176. Model 5, which included only core biomarkers without demographics, showed an AUC of 0.908 (95% CI: 0.825–0.968), an accuracy of 83.8%, a sensitivity of 73.7%, specificity of 87.8, a PPV of 70.0%, an F1 score of 71.8%, and a Brier score of 0.146. Model 6, which included only glial biomarkers without demographics, achieved an AUC of 0.822 (95% CI: 0.703–0.925), an accuracy of 75.0%, sensitivity of 68.4%, specificity of 77.6%, PPV of %54.2, an F1 score of 60.5%, and a Brier score of 0.190. Also, the confusion matrix for the Model 1, and 7 on the independent test set is presented in Supplementary file 1 Table S1. The confusion matrix showed that both Model 1, and 7 retained high sensitivity and specificity despite the class imbalance in the independent test set.

### 3.3 Comparison of discriminative performance

**Figure 2:**
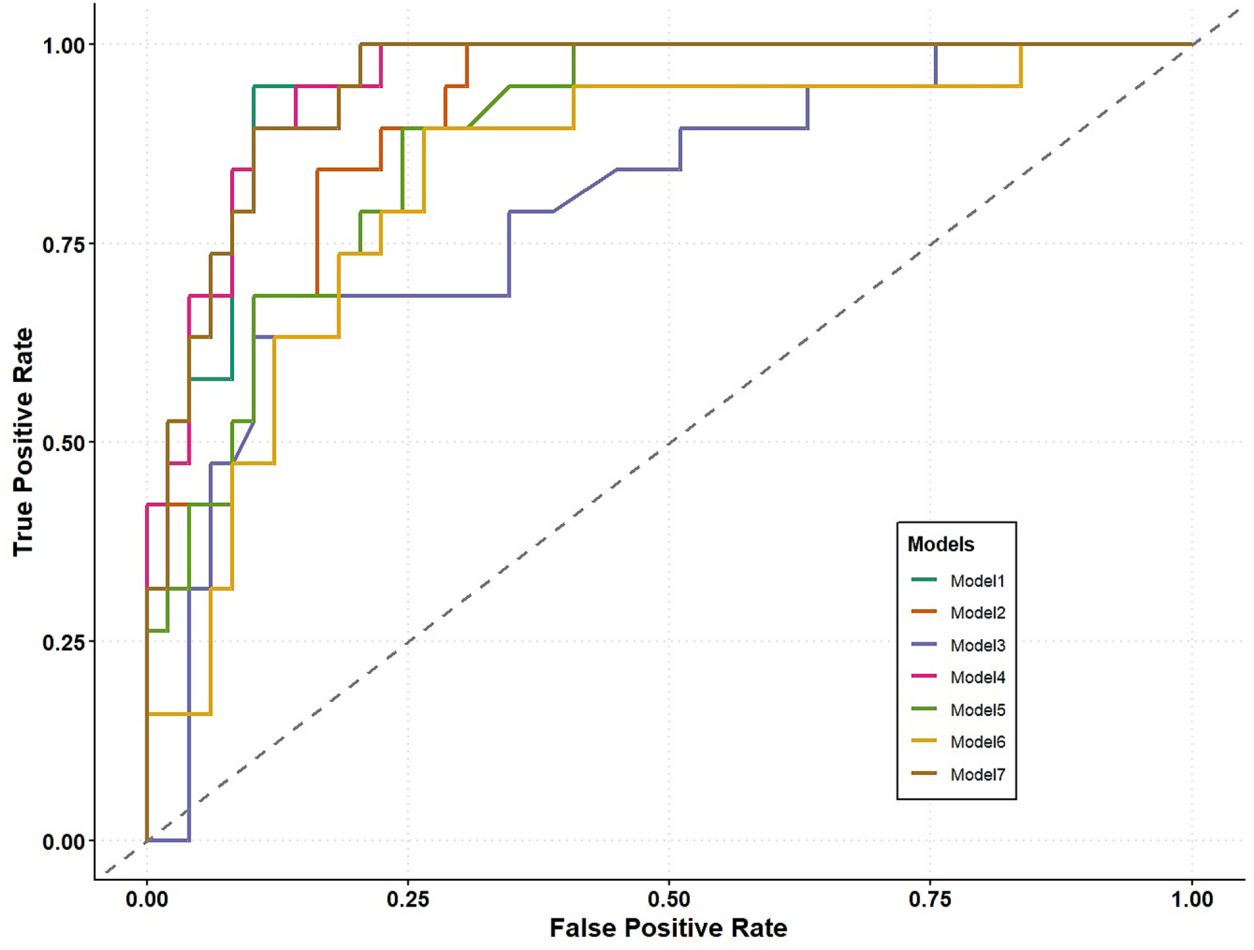
ROC curves for all models.

To thoroughly evaluate the discriminative performance of the models, pairwise comparisons of ROC curves were conducted using DeLong’s test (Table 3) and are shown in Figure 2.

**Table 3:** Pairwise comparison of ROC curves between XGboost models using DeLong’s test.

Comparison (Model A vs. Model B)	AUC _(Model A)_	AUC _(Model B)_	*∆*_AUC_	*p*-value
Model 1vs. Model 2	0.959 (0.908–0.992)	0.888 (0.800–0.955)	0.071	0.003
Model 1vs. Model 3	0.959 (0.908–0.992)	0.830 (0.724–0.919)	0.129	0.011
Model 1vs. Model 4	0.959 (0.908–0.992)	0.955 (0.902–0.990)	0.004	0.340
Model 1vs. Model 5	0.959 (0.908–0.992)	0.908 (0.825–0.968)	0.052	0.035
Model 1vs. Model 6	0.959 (0.908–0.992)	0.822 (0.703–0.925)	0.137	0.016
Model 1vs. Model 7	0.959 (0.908–0.992)	0.951 (0.894–0.989)	0.009	0.309
Model 2 vs. Model 4	0.888 (0.800–0.955)	0.955 (0.902–0.990)	−0.067	0.003
Model 3 vs. Model 4	0.830 (0.724–0.919)	0.955 (0.902–0.990)	−0.125	0.017
Model 3 vs. Model 7	0.830 (0.724–0.919)	0.951 (0.894–0.989)	−0.120	0.023
Model 4 vs. Model 5	0.955 (0.902–0.990)	0.908 (0.825–0.968)	0.047	0.039
Model 4 vs. Model 6	0.955 (0.902–0.990)	0.822 (0.703–0.925)	0.133	0.022
Model 4 vs. Model 7	0.955 (0.902–0.990)	0.951 (0.894–0.989)	0.004	0.539
Model 5 vs. Model 7	0.908 (0.825–0.968)	0.951 (0.894–0.989)	−0.043	0.066
Model 6 vs. Model 7	0.822 (0.703–0.925)	0.951 (0.894–0.989)	−0.129	0.028

Model 1: Core biomarkers + glial biomarkers + age+sex; Model 2: Core biomarkers +age+sex; Model 3: Glial biomarkers + age+sex; Model 4: Core biomarkers + glial biomarkers; Model 5: Core biomarkers; Model 6: Glial biomarkers; Model 7: Hybrid model (LASSO+ XGBoost for all). ∆_AUC_ = AUC _(Model A)_-AUC _(Model B)_; CI: 95% confidence interval with *n* = 2000 bootstrap resampling.

According to DeLong’s test results, Model 1, which includes all biomarkers and demographic variables (AUC = 0.959), did not differ significantly from Model 4, which incorporates core and glial biomarkers (AUC = 0.955, *p* = 0.340), or from the hybrid model (Model 7, AUC = 0.951, *p* = 0.309). In contrast, Model 1 showed significantly higher AUC than the model containing core biomarkers, age, and sex (Model 2, AUC = 0.888, *p* = 0.003); the model containing glial biomarkers, age, and sex (Model 3, AUC = 0.830, *p* = 0.011); the model containing only core biomarkers (Model 5, AUC = 0.908, *p* = 0.035); and the model containing only glial biomarkers (Model 6, AUC = 0.822, *p* = 0.016). Model 4, which combines core and glial biomarkers without incorporating demographic variables (AUC = 0.955), also showed high discriminative performance. Although its AUC value appeared similar to that of Model 2, which included core biomarkers together with age and sex (AUC = 0.888), the difference between the two models was statistically significant (*p* = 0.003). Similarly, the performance of Model 4 was higher than that of the model integrating glial biomarkers with demographics (Model 3; AUC = 0.830; *p* = 0.017), the model using only core biomarkers (Model 5; AUC = 0.908; *p* = 0.039), and the model based solely on glial biomarkers (Model 6; AUC = 0.822; *p* = 0.022).

Model 7, the hybrid model integrating biomarkers selected via LASSO and XGBoost (AUC = 0.951), showed no statistically significant difference in discrimination compared with Model 4 and Model 5 (AUC = 0.955, *p* = 0.539; AUC = 0.908, and *p* = 0.066, respectively). In contrast, Model 7 demonstrated significantly higher AUC values compared with those of Models 2, 3, and 6 (*p* = 0.006, *p* = 0.023, and 0.028, respectively). Pairwise AUC comparisons for all models are provided in Supplementary file 1 Table S2.

### 3.4 SHAP analysis

SHAP analyses were presented in the main manuscript for Model 4, which showed the best performance for biomarker-driven effects without demographics, and for Model 7, a parsimonious model that achieved comparable discriminative performance with fewer predictors. SHAP analyses indicated that both core and glial biomarkers contributed the predictions of both Model 4, and 7.

**Figure 3:**
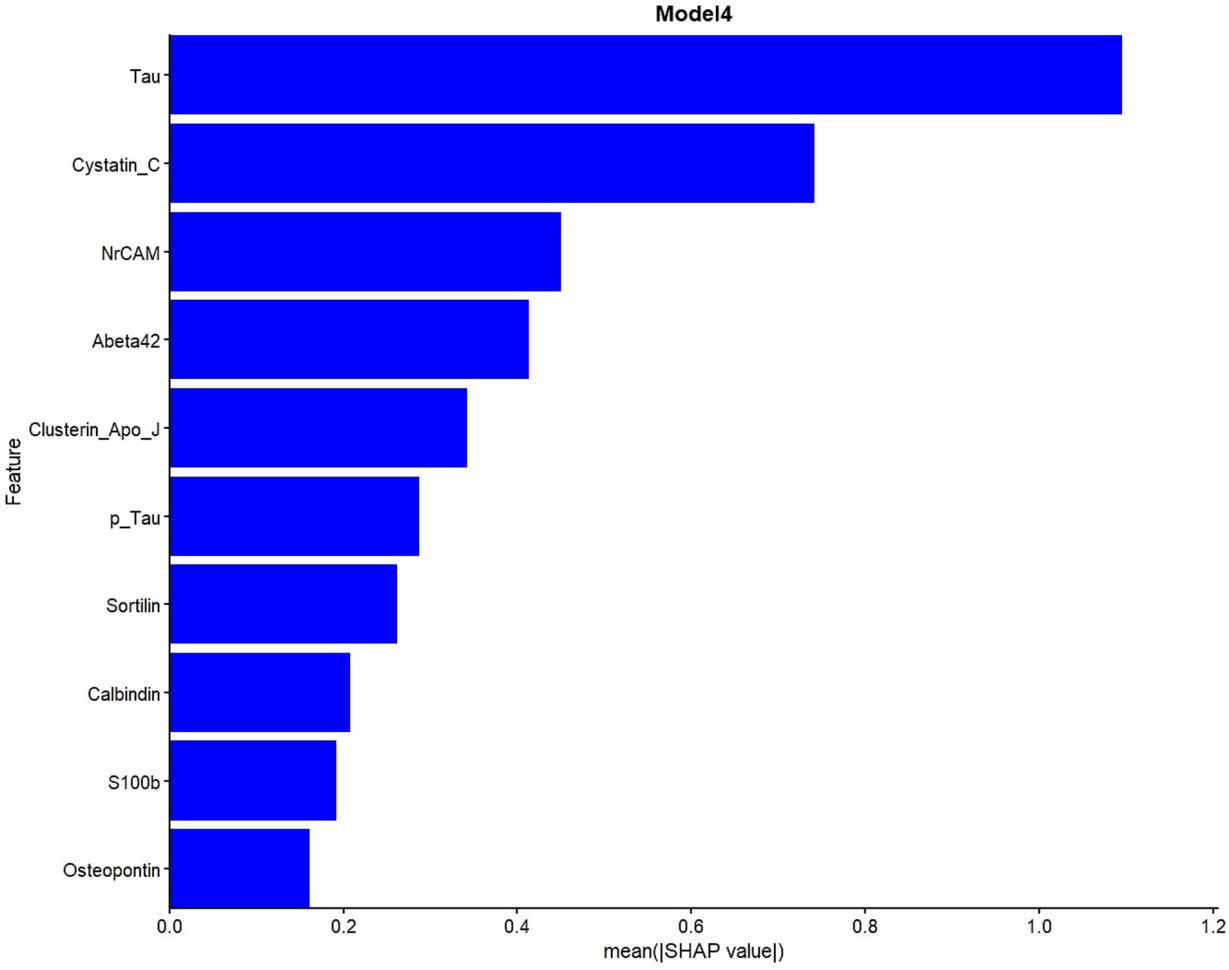
Global SHAP feature importance for Model 4.

**Figure 4:**
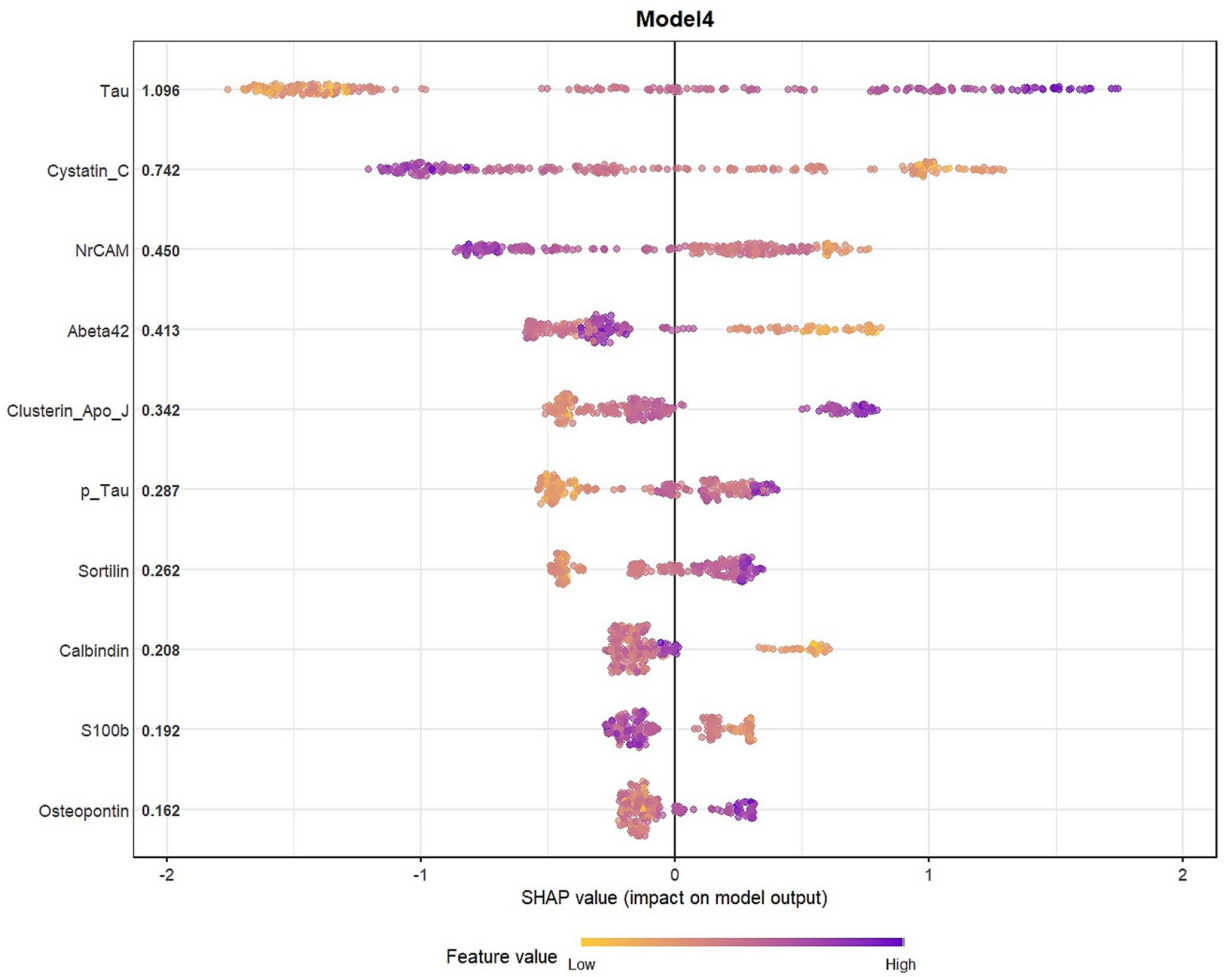
SHAP summary plot for Model 4.

**Figure 5:**
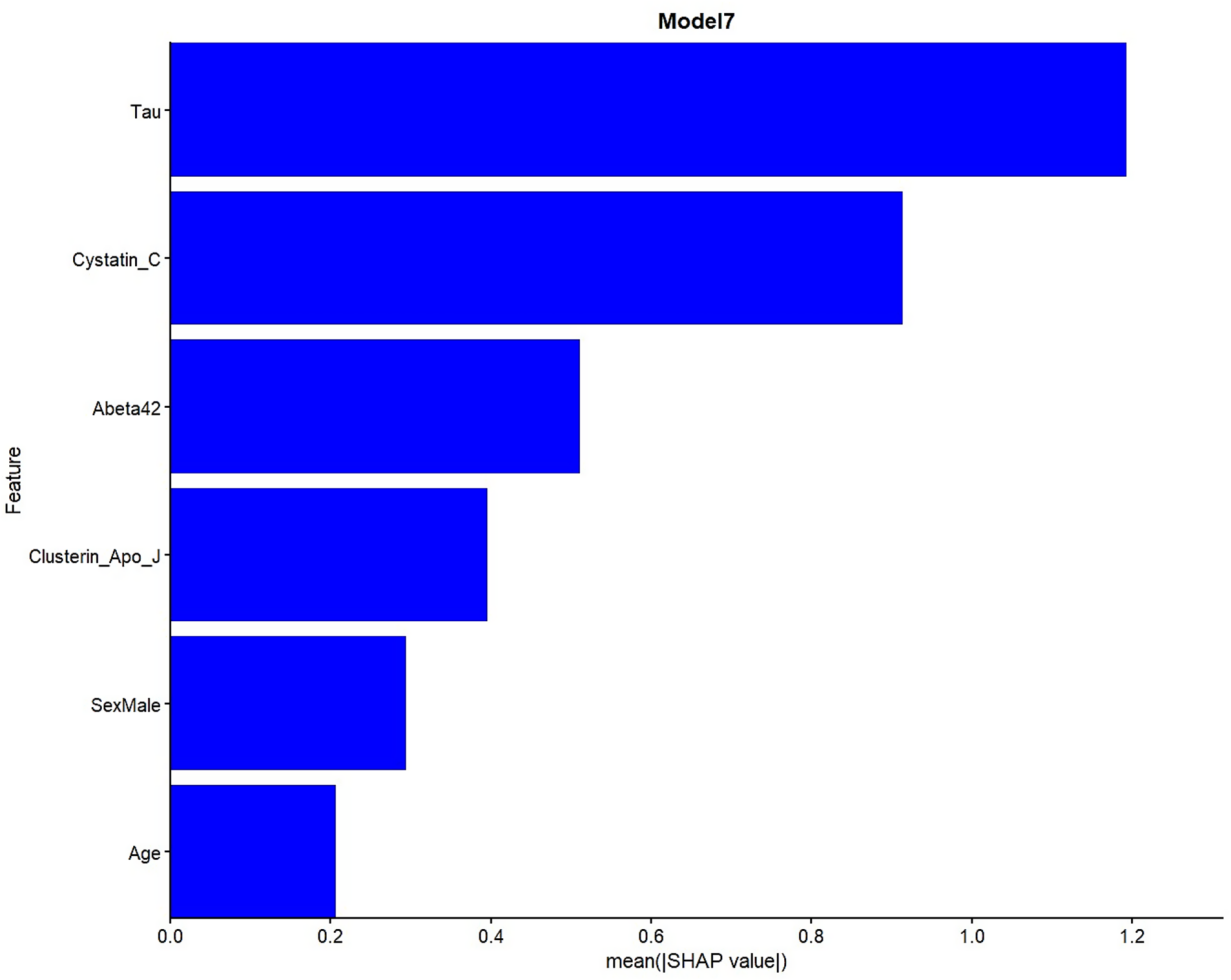
Global SHAP feature importance for Model 7.

Global SHAP feature importance plots show the overall contribution of variables in the model to predictions in a comparative perspective in Figures 3–5. As shown in Figure 3, tau had the highest mean absolute SHAP value, indicating the largest contribution to the model predictions among the included variables. It has been observed that cystatin C and NrCAM showed higher SHAP contributions than Aβ42, while clusterin showed higher SHAP values than p-tau. The contributions of sortilin, calbindin, S100B, and osteopontin were comparatively smaller but remained consistently present across observations.

The concentrated SHAP distributions suggest moderate variability and stable feature contributions across observations. Accordingly, in Figure 4 (Model 4), high tau levels and low cystatin C levels were strongly associated with a higher predicted probability of classification into the impaired group (CDR 0.5–1). Also, other core and glial biomarkers contributed to the model predictions to a moderate extent.

In Model 7, obtained using a simpler combination of variables identified by using LASSO and XGBoost, tau and cystatin C showed the largest SHAP contributions, similar to Model 4. Aβ42 and clusterin showed moderate SHAP contributions, while demographic variables (age, sex) also supported the model predictions (Figure 5).

As tau values increase, SHAP values also tends to increase, suggesting that higher tau values are associated with a higher predicted probability of classification into the “Impaired” class in the model. Low cystatin C values were associated classification into the “Impaired” group, whereas high values associated with predictions toward the control group.

**Figure 6:**
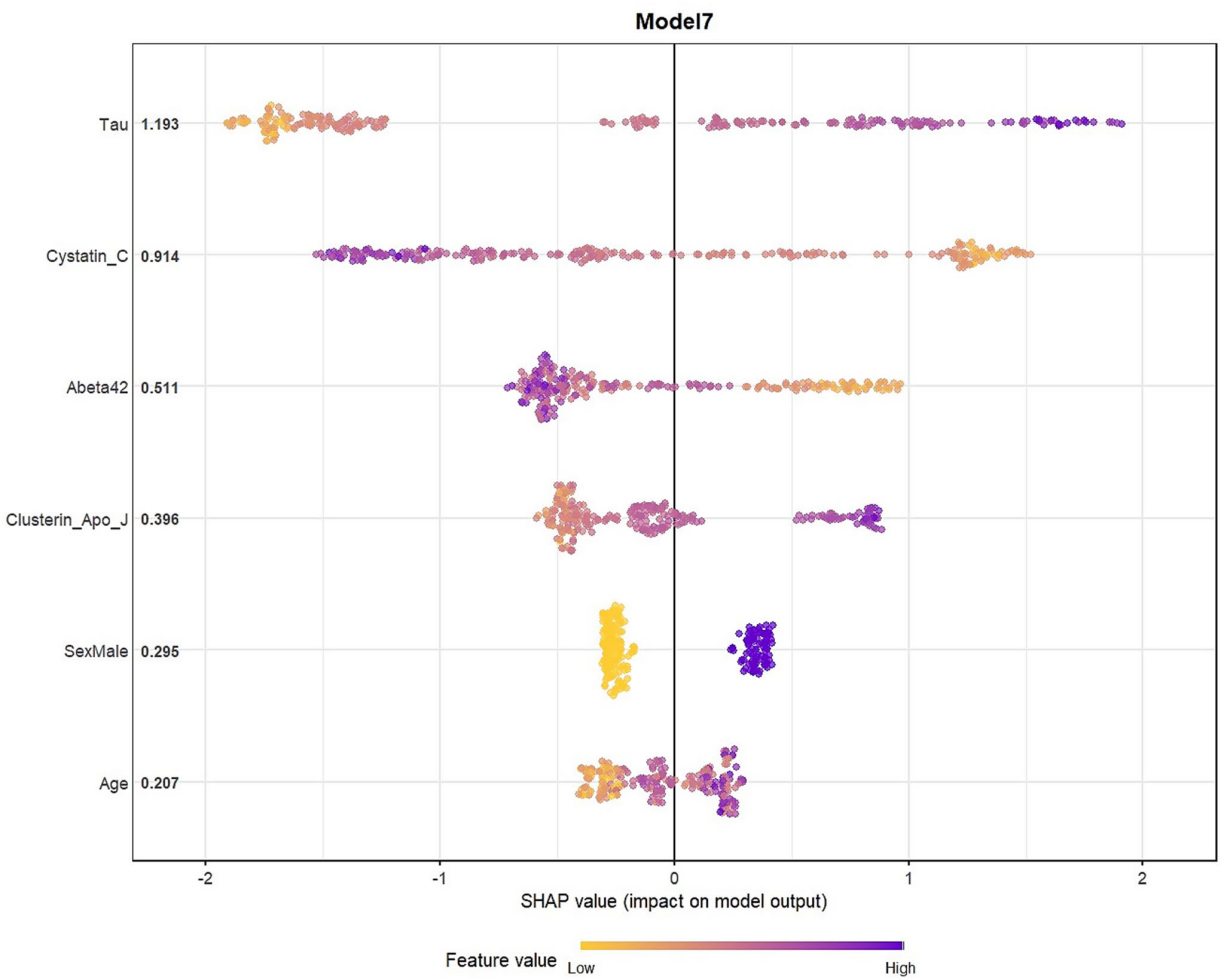
SHAP summary plot for model 7.

As Aβ42 values increased, SHAP values shifted further into the negative region, indicating an association with a lower predicted probability of being classified as impaired in the model. Although the contribution of the glial marker clusterin was low, its higher values shifted the model’s predictions toward AD. Demographic variables also contributed to the model; in particular, male sex and increasing age were associated with higher predicted probabilities of the impaired class (Figure 6). Supplementary file 2 contains SHAP summaries for all other models.

### 3.5 Evaluation of model validity and assessment of overfitting

**Figure 7:**
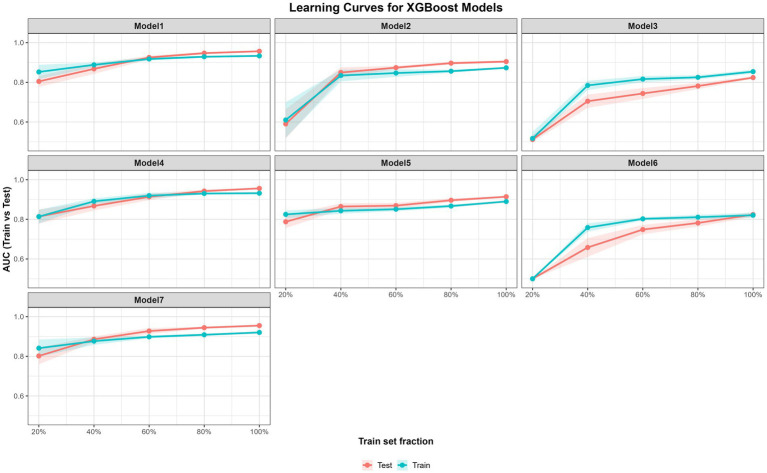
AUC-based learning curves for all XGBoost models.

AUC-based learning-curves were used to assess model stability and potential overfitting across increasing training fractions (Figure 7). As the training data proportion increased, the AUC values for both training and test sets gradually converged for most models. The train-test AUC differences (GAP = *AUC_train_–AUC_test_*) were generally minimal at higher training fractions. For the best-performing models (Models 1, 4, and 7), GAP values ranged between −0.035 and −0.023 at the full training fraction, indicating that the test performance was comparable to the training results (Supplementary file 1 Table S3). This pattern suggests limited training-set optimism and supports the stability of the models.

Models based solely on core or glial biomarkers showed lower overall AUC values; however, their learning curves also showed decreasing train-test differences as the training size increased. Although moderate GAP values were observed at smaller training fractions (e.g., Model 6: GAP = 0.100 at fraction = 0.4), these differences diminished with larger training sizes. Overall, the convergence of training and test AUC values at higher training fractions is consistent with stable model behavior and does not suggest substantial overfitting in the evaluated models.

In addition to discrimination stability, model validity was assessed through calibration analysis on the independent test set. Models 1, 4, and 7 demonstrated Brier scores of approximately 0.11, which represent a meaningful improvement over the prevalence-based baseline (0.198) and reflected favorable probabilistic accuracy. Calibration curves (Supplementary file 1 Figure S11) showed that the predicted probabilities were generally aligned with observed outcomes, although calibration slopes greater than 1 (ranging from 1.49 to 2.08) were observed. Collectively, the convergence of AUC values and the probabilistic improvements were indicated by the Brier scores suggest that the models provide a reliable framework for classification without evidence of substantial overfitting.

### 3.6 Stability analysis of LASSO-based feature selection

The stability analysis of LASSO-based feature selection showed that several predictors were consistently retained across repeated resampling iterations (Supplementary file 1 Table S4). Aβ42, tau, age, and sex were selected in 100% of the iterations, while Cystatin C was selected in 99.6% of iterations, reflecting very high selection stability for these predictors. These variables correspond to the biomarkers retained in the hybrid model and form the core predictor set of the model. Among the remaining biomarkers, Clusterin Apo-J showed moderate selection stability, being selected in 63.6% of the iterations. In contrast, other glial biomarkers showed lower selection frequencies, including Sortilin (31.8%) and NrCAM (25.5%). Several biomarkers were selected only rarely across the resampling iterations, such as S100b (2.0%), p-tau (1.0%), Calbindin (0.4%), and Osteopontin (0.2%).

## 4 Discussion

This study indicates that integrating glial activation biomarkers with traditional CSF core biomarkers within an ML framework enhances the classification performance for distinguishing cognitively unimpaired individuals from those with very mild or mild clinical impairment. In this study, we applied an advanced ML framework that combined hybrid feature selection with the XGBoost-SHAP algorithm to evaluate the measurable and clinically meaningful contribution of adding glial activation biomarkers to established AD-related core CSF biomarkers for the classification of CDR-defined CI. Seven predictive models were developed in different combinations. The hybrid approach combines LASSO’s variable reduction capability with XGBoost’s strong classification performance, yielding a parsimonious model that retains only the most informative biomarkers.

Our findings emphasize that potential role of integrating multiple biomarker domains for accurate classification of CDR-defined clinical status. Across models, our results consistently show that various combinations of core and glial biomarkers (Models 1, 4, and 7) achieved the highest classification performance, suggesting that both neuronal and glial pathways are involved in biological processes linked to AD, and that integrating these dimensions improves classification of CDR-defined clinical CI. Notably, no significant difference was observed in the classification performance of these models (*p* > 0.05, DeLong test). Although demographic variables increased the classification performance of Models 1 and 7, the performance of Model 4 did not change when these variables were removed from the model. Similar results were observed with different combinations of traditional core biomarkers. Model 2 (established with core biomarkers, age, and sex; AUC = 0.888) and Model 5 (established with core biomarkers only; AUC = 0.908) showed similarly high performance. It showed us the added value of demographic variables may be limited when high-performing biomarkers are already present. The minimal performance gain implies that, although age and sex are well-known risk factors, their discriminative contribution is secondary to that of core biomarkers in classification tasks. From a clinical perspective, this finding implies that even without individualized demographic profiling, integrated biomarker signatures may allow reliable early-stage clinical classification. Consistent with the learning-curve analysis, the small train-test AUC differences observed for the best-performing models had limited training-set optimism. However, the modest sample size and single-center design indicate that external validation in independent cohorts will be necessary before clinical application. Also, the stability analysis showed that the key predictors included in the hybrid model (Aβ42, tau, age, sex, and Cystatin C) were consistently selected across repeated resampling iterations, supporting the robustness of the core biomarker structure identified by the LASSO-based selection approach.

Models based on a single biomarker group consistently performed poorly predictive performance. CI associated with AD-related biological processes tended to be multidimensional and may not be adequately understood through a single biological axis. Although the classification accuracy of Model 6, composed of glial biomarkers, remains at a reasonable level (accuracy = 0.750), the model’s high discriminant power (AUC = 0.822) demonstrates that glial activation can carry biological signals relevant to CDR-defined CI, even when evaluated independently. Glial biomarkers alone reflect a meaningful relationship with the CI, but when combined with other biomarkers (Model 1 and Model 4), classification performance is maximized. This supports accumulating evidence that glial dysregulation is an early and independent component of AD-related pathophysiology, detectable before substantial neuronal degeneration. These results align with recent evidence suggesting that multivariate biomarker integration enhances diagnostic accuracy and may provide a more robust foundation for clinical decision-support tools. Current studies show that glial biomarkers, particularly glial fibrillary acidic protein (GFAP) for astrocytes and sTREM2, YKL-40 for microglia, are increasingly recognized as important biomarkers in AD diagnosis and when combined with core AD biomarkers, improve diagnostic sensitivity and specificity (32). While those studies focus on biological AD diagnosis, our findings extend this perspective by demonstrating that glial biomarkers are also selected as key predictors in models classifying CDR-defined clinical cognitive impairment.

The hybrid model selected the variables LASSO, clusterin, cystatin C, age, tau, Aβ42, and sex as key predictors for the classification of CDR-defined clinical CI. Notably, parsimonious approach achieved classification performance comparable to Models 1 and 4, which utilize larger biomarker panels. This suggests that a streamlined set of markers can maintain strong classification capacity while enhancing model interpretability. Furthermore, the inclusion of glial biomarkers in the final selection supports their potential contribution to improving the classification of CDR-defined clinical status within this research context.

The evaluation of the confusion matrix on the independent test set indicated that threshold optimization effectively repositioned the decision boundary to fit the data distribution without altering the model’s discriminatory power. Despite the inherent class imbalance in the test set (49 controls vs. 19 impaired), both Model 1, and 7 demonstrated balanced sensitivity and specificity. Specifically, Model 7—a primary focus of this study—correctly classified 17 out of 19 cognitively impaired individuals with a low rate of false negatives. These findings suggested that smaller, more interpretable biomarker panels may offer performance levels similar to more complex models in distinguishing clinical cognitive status. This stability was further supported by the learning curve analysis, where the convergence of training and test AUC values at larger training fractions points toward stable model behavior without clear evidence of substantial overfitting.

The calibration analysis showed that integrating multiple biomarker domains enhances the reliability of clinical stratification beyond simple prevalence-based expectations. According to our results, the combined panels provided more refined probability estimates, as evidenced by the consistent alignment between predicted and observed outcomes in the reliability plots. While these results underscore the potential of multidimensional biomarker integration in characterizing clinical status, the observed calibration patterns also highlight the importance of further refining these estimates. Therefore, external validation in larger, independent cohorts remains an essential step to confirm the generalizability of these probabilistic models. From a translational perspective, this parsimonious hybrid model may offer a feasible approach by reducing the number of required biomarkers while maintaining classification accuracy for CDR-defined clinical cognitive impairment. Such a model could potentially be integrated into CSF-based workflows in memory clinics as a risk stratification tool, particularly in early-stage or diagnostically ambiguous cases.

SHAP analysis revealed that tau and cystatin C were the most influential predictors within our models, while core and glial biomarkers formed a hierarchical structure shaping classification of CDR-defined clinical cognitive impairment. Specifically, high tau levels and low cystatin C levels were consistently associated with classification into the impaired group, whereas increased Aβ42 linked to a reduced likelihood such classification. Importantly, the hybrid model (Model 7), reproduced similar effect patterns despite using fewer predictors, highlighting the potential for parsimonious marker panel can still capture strong biological signals. These findings point to the importance of both core and glial biomarkers in determining CDR-defined clinical status, offering a perspective where glial measurements may play a more prominent role in future diagnostic models. Osteopontin and clusterin were also prominent in the selected models, further supporting the view that neuroinflammatory signaling provides diagnostic information not fully captured by amyloid or tau pathology alone. The variability in Aβ_42_’s relative contribution across models aligns with previous observations that amyloid burden, although central to AD pathophysiology, shows limited incremental value for clinical discrimination. Cystatin C is a cysteine protease inhibitor highly expressed in the brain and CSF, produced by neurons, astrocytes, and microglia and capable of binding Aβ to inhibit its aggregation and fibril formation while protecting neurons from Aβ-induced toxicity (33). CSF cystatin C levels are often reduced in AD compared to controls, and lower levels are also seen in other dementias with amyloid pathology (34). However, some studies report no significant difference or even conflicting results (35). Although CSF cystatin C shows heterogeneous results across studies, its functional role in amyloid processing and neuroprotection may help explain why it emerged as a strong predictor across multiple models. Clusterin, also known as apolipoprotein J, is predominantly synthesized and secreted in the cerebral parenchyma by astrocytes (36). Clinically, plasma clusterin has been associated with AD disease severity and progression. For example, higher plasma clusterin correlated with greater brain atrophy and more rapid cognitive decline in AD patients (37). In patients with MCI, elevated clusterin conferred increased risk of conversion to AD and faster decline (38). Interestingly, clusterin did not differ between AD patients and controls in that study, but it did correlate with core AD biomarkers, including tau and p-tau. Osteopontin is a multifunctional, proinflammatory glycoprotein secreted by microglia, astrocytes, and immune cells in response to injury and stress (39). In AD, osteopontin is upregulated in the CSF and plasma, and is involved in neuroinflammation, microglial activation, and Aβ clearance (40, 41). Its levels are elevated early, correlate with pathology and cognitive decline, and may help monitor disease course (42). Their prominence in several SHAP profiles, similar to cystatin C, supports the notion that glial-derived signals capture additional dimensions associated with AD-related biology that extend beyond amyloid or tau biomarkers. In the context of our study, this provides a mechanistic rationale for their contribution to classification of CDR-defined clinical CI in multivariate ML models.

Together, these findings indicate that glial-derived biomarkers encode biologically complementary information that can enhance model performance, reinforcing the rationale for extending the AT(N) framework to incorporate an “I” (inflammation) component. The robustness of glial feature importance across diverse modeling approaches further underscores their potential utility in multidimensional biomarker panels aimed at improving classification of CDR-defined clinical CI.

The widespread use and ease interpretation of AI and ML have also popularized AD prediction studies that use multimodal biomarkers. These methods play an important role in creating new models with comprehensive and multi-modal data sets, identifying potential biomarkers, and in obtaining accurate results from them. Neuroimaging, demographic and clinical characteristics, neuropsychological test scores (43, 44). Prediction models created using different combinations of multimodal biomarkers, including CSF biomarkers, and APOE e4 genotype have been shown to yield more accurate, robust, and reliable predictions of conversion from MCI to AD (45). Several common features of these studies are their frequent use of noninvasive assessment tools and their achieving the highest predicted values using the XGBoost method, similar to our findings. For example, a recent study by Jiao et al. reported high diagnostic performance (AUC 0.92–0.94) for biomarker panels integrating p-tau, Aβ_42_, and GFAP in pathology-defined AD cohorts (46). In contrast, other plasma-based studies have shown that although GFAP and related glial biomarkers correlate with cognitive scores, they do not significantly enhance clinical staging stratification (47). Within this context, our findings suggest that selected CSF-derived glial biomarkers, such as clusterin and cystatin C, may retain meaningful discriminative relevance for CDR-defined clinical impairment when integrated into a multivariate ML framework.

Beyond multimodal integration, another key theme in recent ML-based dementia research is the development of parsimonious models that maintain high performance with fewer variables. In a previous study comparing ML methods using 70 features, including cognitive, socioeconomic, clinical, and imaging variables, LASSO identified a reduced subset of 17 key predictors, achieving the highest accuracy, followed closely by XGBoost (48). Consistent with this principle, our hybrid model created using LASSO showed performance comparable to these results. Using this model, we derived a classification model with high accuracy and strong performance that required fewer variables. After applying LASSO+XGBoost to Model 1, which we created with 12 variables including core, glial, and clinical features, the method selected clusterin, cystatin C, tau, Aβ_42_, sex, and age, reducing the number of variables to 6. The model accuracy (accuracy = 0.838) was equivalent to that of Model 1, and its classification performance was similarly successful. Achieving the same classification performance with fewer biomarkers, rather than using more biomarkers, was an important finding of our study.

While current plasma-based panels using p-tau and Aβ_42/40_ ratio provide high sensitivity and specificity (often >90%) for AD pathology, closely mirroring CSF and PET results (49, 50). They primarily reflect amyloid and tau load, and may not fully capture the inflammatory and neuroimmune components that are increasingly recognized as early and independent drivers of disease progression. Our study adds value by incorporating glial biomarkers such as clusterin, cystatin C, and osteopontin, which reflect astrocytic and microglial responses and provide mechanistic insights beyond classical proteinopathies. Moreover, our findings suggested that the selection of glial biomarkers contribute comparably to established core biomarkers, including tau, in multivariate classification models, and that a small hybrid panel (e.g., tau, Aβ_42_, cystatin C, and clusterin) could achieve similar classification performance while providing potentially greater biological diversity. This not only reinforces the rationale for the proposed AT(N)I framework but also supports the idea that diverse pathophysiological processes can be captured with a compact, yet complementary marker set.

Our findings are in line with a growing body of research that supports the use of ML to uncover meaningful diagnostic signals in glia-related biomarkers. A study employed multiplex proximity extension assays (PEA) to quantify neuroinflammation-related proteins in CSF, including biomarkers of astrocyte and microglial activation. In this study, using penalized logistic regression identified SIRT2, HGF, MMP-10, and CXCL5 as top discriminators between AD and controls, with these proteins linked to glial activation and blood–brain barrier dysfunction (51). Another study applied cyclic multiplex fluorescent immunohistochemistry to human brain tissue, measuring multiple astroglial (e.g., GFAP, YKL-40) and microglial (e.g., IBA1, CD68, TMEM119) biomarkers. ML models (gradient boosting, CNNs) accurately distinguished AD from controls at the single-cell level based on glial marker profiles (52). Moreover, ML was used to segment and analyze microglia in retinal tissue, revealing differences in the number, size, and CD68 + activation of microglia between AD and controls (53). Studies have used single-cell RNA sequencing and ML to identify microglial subtypes and gene signatures associated with AD, thereby supporting the diagnostic value of glial biomarkers (54).

This study has some limitations. We were conducted using an open-access dataset. Although the use of open data enhances reproducibility, the lack of control over data collection processes and heterogeneity of clinical variables remains a significant limitation. Because the publicly available dataset combines individuals with CDR 0.5 and CDR 1 into a single “Impaired” category, subgroup-specific model performance across impairment severity levels could not be evaluated and separate severity labels are not accessible. In addition, the dataset provides only CDR-based clinical groupings (Control vs. Impaired) without biomarker-defined AD pathology status; therefore, the cohort likely represents a pathology-heterogeneous population and the models should be interpreted as classifiers of clinical CI rather than biomarker confirmed AD. Therefore, future studies using datasets with granular CDR stratification are warranted.

The relatively small sample size, particularly within the impaired group, combined with the single-center nature of the dataset may limit the direct generalizability of our findings. While we carefully implemented internal validation procedures—including a held-out test set, bootstrap-based uncertainty estimation, and learning-curve analysis—these methods serve to demonstrate model stability rather than substitute for true external validation. Consequently, our results should be interpreted as internally validated findings within this specific research context. Further multi-center studies using independent cohorts are essential to confirm these results and ensure their reliability before any potential clinical application. Although the hybrid modeling approach produces robust results, different algorithms or penalized strategies can generate alternative sets of predictors.

## 5 Conclusion

In summary, while glial biomarkers have been extensively studied in neurodegenerative research, growing evidence supports their clinical relevance when integrated into multidimensional frameworks. Our findings align with this perspective, demonstrating that the combination of glial and traditional core CSF biomarkers provides a more comprehensive profile of clinical cognitive status. Specifically, our results illustrate that even a parsimonious, strategically selected subset of these biomarkers can maintain high classification performance for CDR-defined impairment. Rather than providing a definitive diagnosis of Alzheimer’s pathology, this interpretable ML approach offers a promising framework for scalable risk stratification within research and clinical research settings.

## Data Availability

The data used in this study are publicly available and can be accessed through the Comprehensive R Archive Network (CRAN). Specifically, the dataset is distributed within the AppliedPredictiveModeling R package, which accompanies the predictive modeling examples presented by Kuhn and Johnson (26). These data are a modified version of the values used for the publication. The dataset is available under the name “AlzheimerDisease” and includes predictors (biomarker measurements) and diagnostic class labels used for prediction in Alzheimer’s disease classification studies. The dataset is not distributed as a standalone file but can be accessed directly within the R statistical environment after installing the AppliedPredictiveModeling package using the following commands: install.packages(“AppliedPredictiveModeling”), followed by: library(AppliedPredictiveModeling), and: data(“AlzheimerDisease”).
