# Supplementary material for: Glial biomarkers improve classification of cognitive impairment: an explainable artificial intelligence study using CSF biomarkers
Source: Front Neurol. 2026 Apr 23;17:1787915. doi: 10.3389/fneur.2026.1787915 (PMC13149148; doi:10.3389/fneur.2026.1787915)
Supplement: Supplementary file 1 [file Data_Sheet_1.pdf]

Table S1. Confusion matrix for Model 1 and the Model 7

		**Predicted**	
**Actual**	**Model 1 (Test set 20%)**	Impaired (+)	Control (-)
	Impaired (+)	17	2
	Control (-)	4	45
	**Model 7 (Test set 20%)**		
	Impaired (+)	16	3
	Control (-)	6	43

Model 1: Core markers + glial markers + age +sex; **Model 7:** Hybrid model (LASSO+ XGBoost for all markers). Positive class is accepted as impaired group. Confusion matrices were computed on the held-out test set using the ROC-optimized threshold (thresholds Model 1: 0.166; Model 7: 0.173, respectively) and evaluated on the independent test set (20% split).

Table S2: DeLong test results for pairwise AUC comparisons among models

		AUC (95% CI)			
**Model A**	**Model B**	**Model A**	**Model B**	Δ_{AUC}	***p*-value**
Model 1	Model 2	0.959 (0.908-0.992)	0.888 (0.800-0.955)	0.071	0.003
Model 1	Model 3	0.959 (0.908-0.992)	0.830 (0.724-0.919)	0.129	0.011
Model 1	Model 4	0.959 (0.908-0.992)	0.955 (0.902-0.990)	0.004	0.340
Model 1	Model 5	0.959 (0.908-0.992)	0.908 (0.825-0.968)	0.052	0.035
Model 1	Model 6	0.959 (0.908-0.992)	0.822 (0.703-0.925)	0.137	0.016
Model 1	Model 7	0.959 (0.908-0.992)	0.951 (0.894-0.989)	0.009	0.309
Model 2	Model 3	0.888 (0.800-0.955)	0.830 (0.724-0.919)	0.057	0.355
Model 2	Model 4	0.888 (0.800-0.955)	0.955 (0.902-0.990)	-0.067	0.003
Model 2	Model 5	0.888 (0.800-0.955)	0.908 (0.825-0.968)	-0.020	0.181
Model 2	Model 6	0.888 (0.800-0.955)	0.822 (0.703-0.925)	0.066	0.335
Model 2	Model 7	0.888 (0.800-0.955)	0.951 (0.894-0.989)	-0.063	0.006
Model 3	Model 4	0.830 (0.724-0.919)	0.955 (0.902-0.990)	-0.125	0.017
Model 3	Model 5	0.830 (0.724-0.919)	0.908 (0.825-0.968)	-0.077	0.218
Model 3	Model 6	0.830 (0.724-0.919)	0.822 (0.703-0.925)	0.009	0.817
Model 3	Model 7	0.830 (0.724-0.919)	0.951 (0.894-0.989)	-0.120	0.023
Model 4	Model 5	0.955 (0.902-0.990)	0.908 (0.825-0.968)	0.047	0.039
Model 4	Model 6	0.955 (0.902-0.990)	0.822 (0.703-0.925)	0.133	0.022
Model 4	Model 7	0.955 (0.902-0.990)	0.951 (0.894-0.989)	0.004	0.539
Model 5	Model 6	0.908 (0.825-0.968)	0.822 (0.703-0.925)	0.086	0.213
Model 5	Model 7	0.908 (0.825-0.968)	0.951 (0.894-0.989)	-0.043	0.066
Model 6	Model 7	0.822 (0.703-0.925)	0.951 (0.894-0.989)	-0.129	0.028

Model 1: Core markers + glial markers + age+sex; **Model 2:** Core markers +age+sex; **Model 3:** Glial markers + age+sex; **Model 4:** Core markers + glial markers; **Model 5:** Core markers; **Model 6:** Glial markers; **Model 7:** Hybrid model (LASSO+ XGBoost for all). Δ_{AUC}= $AUC_{model\ A}$-$AUC_{model\ B}$ **C.I.**: 95% confidence interval with n=2000 bootstrap re-sampling.

Table S3. GAP values Learning curves

		AUC (95% CI)		
**Models**	**Training set fraction**	**Test data set**	**Train data set**	**GAP**
Model1	0.200	0.805 (0.779-0.830)	0.852 (0.819-0.888)	0.048
Model1	0.400	0.868 (0.845-0.893)	0.888 (0.878-0.901)	0.020
Model1	0.600	0.926 (0.917-0.934)	0.918 (0.911-0.925)	-0.008
Model1	0.800	0.947 (0.943-0.953)	0.929 (0.925-0.934)	-0.018
Model1	1.000	0.957 (0.956-0.958)	0.933 (0.932-0.934)	-0.023
Model2	0.200	0.590 (0.523-0.663)	0.610 (0.527-0.695)	0.020
Model2	0.400	0.849 (0.821-0.870)	0.834 (0.808-0.864)	-0.015
Model2	0.600	0.874 (0.866-0.882)	0.847 (0.828-0.865)	-0.027
Model2	0.800	0.897 (0.890-0.904)	0.856 (0.849-0.864)	-0.041
Model2	1.000	0.905 (0.899-0.910)	0.873 (0.870-0.876)	-0.031
Model3	0.200	0.511 (0.500-0.534)	0.517 (0.500-0.551)	0.005
Model3	0.400	0.705 (0.673-0.736)	0.785 (0.763-0.807)	0.080
Model3	0.600	0.744 (0.714-0.767)	0.816 (0.802-0.832)	0.073
Model3	0.800	0.781 (0.768-0.796)	0.825 (0.816-0.835)	0.044
Model3	1.000	0.824 (0.818-0.830)	0.854 (0.844-0.862)	0.030
Model4	0.200	0.814 (0.779-0.846)	0.814 (0.781-0.845)	0.000
Model4	0.400	0.868 (0.846-0.889)	0.891 (0.878-0.905)	0.023
Model4	0.600	0.914 (0.897-0.929)	0.919 (0.907-0.933)	0.006
Model4	0.800	0.943 (0.936-0.949)	0.930 (0.925-0.936)	-0.012
Model4	1.000	0.956 (0.955-0.957)	0.932 (0.931-0.932)	-0.024
Model5	0.200	0.788 (0.758-0.816)	0.825 (0.806-0.843)	0.037
Model5	0.400	0.865 (0.848-0.879)	0.843 (0.829-0.856)	-0.021
Model5	0.600	0.869 (0.857-0.884)	0.851 (0.842-0.860)	-0.018
Model5	0.800	0.896 (0.888-0.906)	0.867 (0.859-0.875)	-0.029
Model5	1.000	0.914 (0.912-0.916)	0.890 (0.889-0.891)	-0.024
Model6	0.200	0.500 (0.500-0.500)	0.500 (0.500-0.500)	0.000
Model6	0.400	0.658 (0.612-0.701)	0.758 (0.737-0.777)	0.100
Model6	0.600	0.748 (0.726-0.769)	0.802 (0.796-0.810)	0.054
Model6	0.800	0.782 (0.765-0.799)	0.811 (0.801-0.822)	0.029
Model6	1.000	0.824 (0.812-0.834)	0.821 (0.809-0.833)	-0.003
Model7	0.200	0.802 (0.762-0.841)	0.841 (0.801-0.882)	0.039
Model7	0.400	0.886 (0.872-0.899)	0.877 (0.859-0.895)	-0.009
Model7	0.600	0.927 (0.912-0.939)	0.898 (0.891-0.905)	-0.029
Model7	0.800	0.945 (0.940-0.949)	0.909 (0.903-0.915)	-0.036
Model7	1.000	0.955 (0.954-0.956)	0.920 (0.920-0.921)	-0.035

95% confidence intervals (CI) were computed with repetition-based CI.

Table S4. Selection frequency of biomarkers across repeated LASSO subsampling iterations (500 iterations)

		**N (500)**	**Selection frequency (%)**
Aβ42		500	100
Tau		500	100
Age		500	100
Sex		500	100
Cystatin c		498	99.6
Clusterin Apo J		318	31.8
Sortilin		159	26.8
NrCAM		134	25.5
S100b		10	2
p-tau		5	1
Calbindin		2	0.4
Osteopontin		1	0.2

Selection frequencies represent the proportion of iterations in which each biomarker was retained by the LASSO model using the *lambda1.se* criterion across 500 repeated stratified subsampling iterations (80% of the training data per iteration).

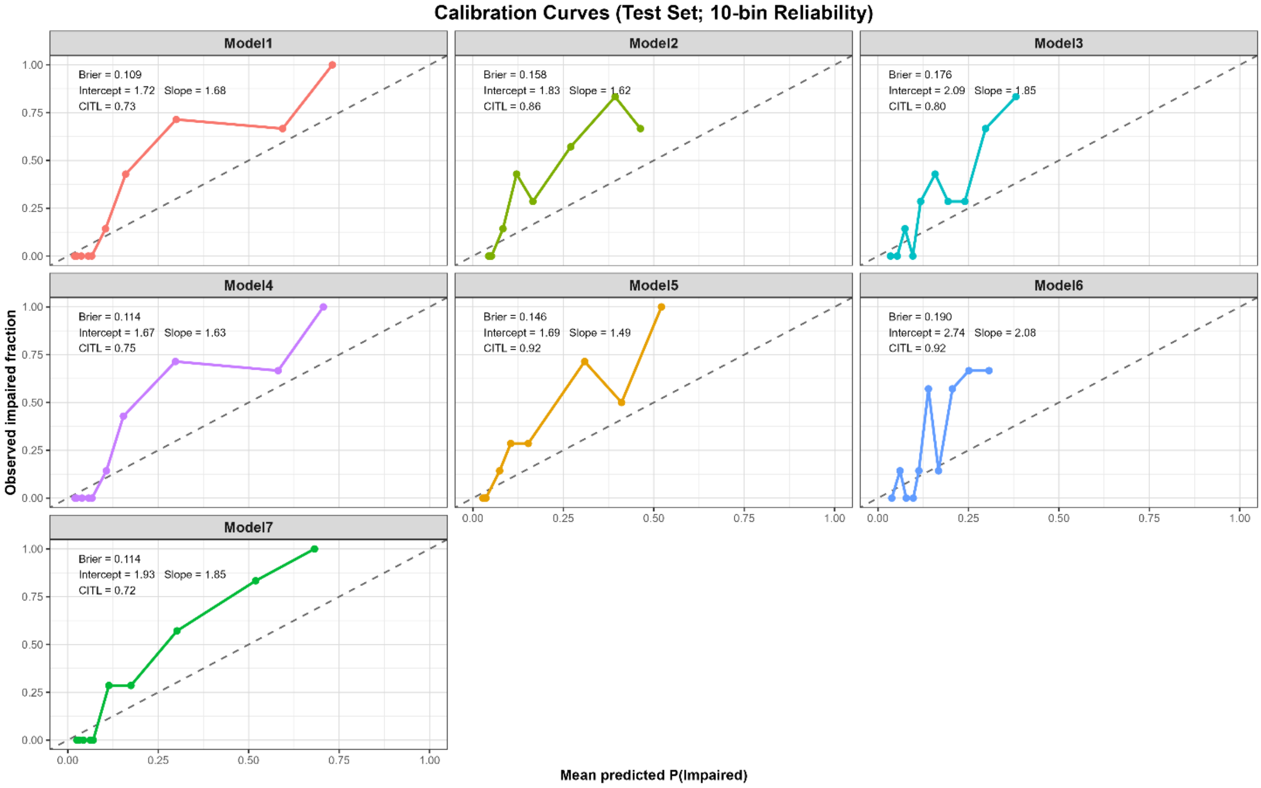

Figure S11: Calibration of machine learning models for cognitive impairment classification

Calibration curves for the seven predictive models on the independent test set (n=68). Each panel displays the relationship between the mean predicted probability and the observed frequency of cognitive impairment (CDR 0.5 or 1). The dashed diagonal line represents perfect calibration (Ideal).
