# Supplementary material for: Glial biomarkers improve classification of cognitive impairment: an explainable artificial intelligence study using CSF biomarkers
Source: Front Neurol. 2026 Apr 23;17:1787915. doi: 10.3389/fneur.2026.1787915 (PMC13149148; doi:10.3389/fneur.2026.1787915)
Supplement: Supplementary file 2 [file Data_Sheet_2.pdf]

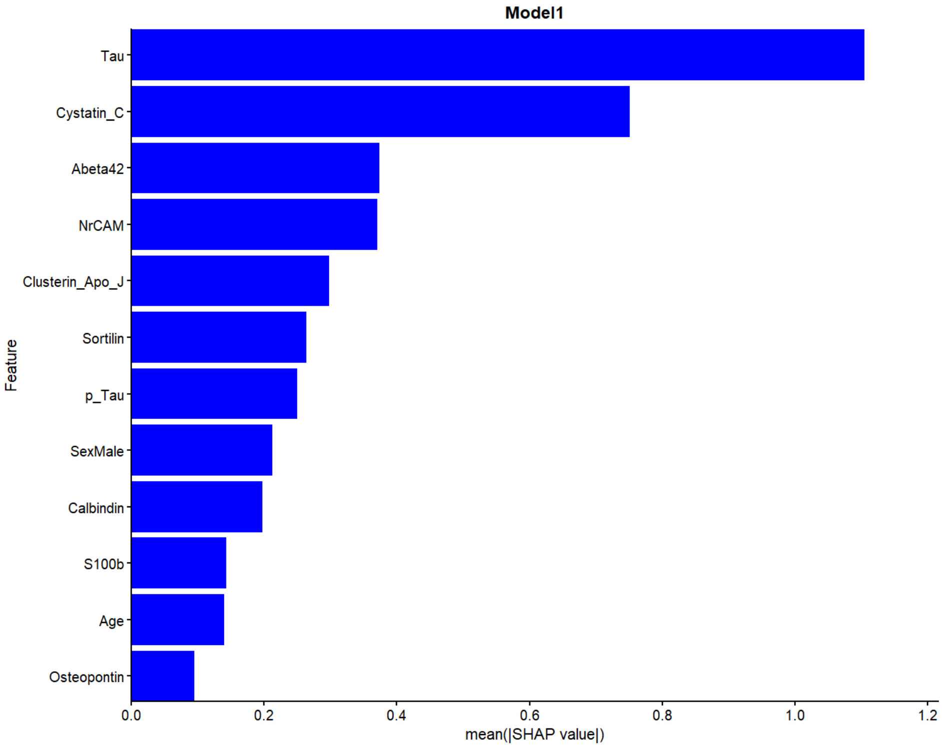

Figure S1. Global SHAP feature importance for model 1

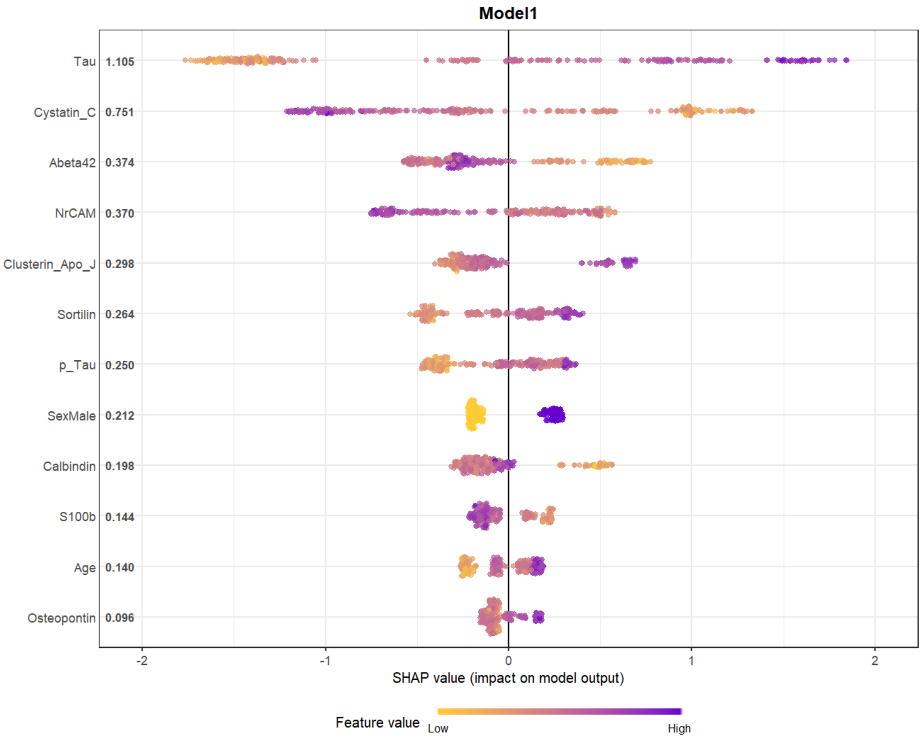

Figure S2. SHAP summary plot for Model 1

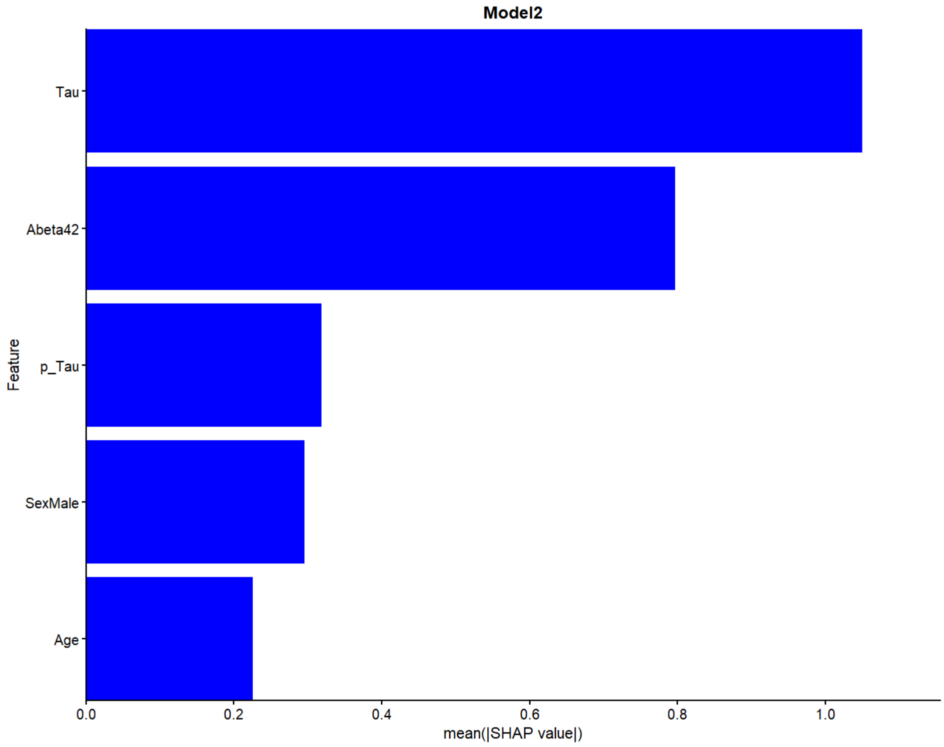

Figure S3. Global SHAP feature importance for model 2

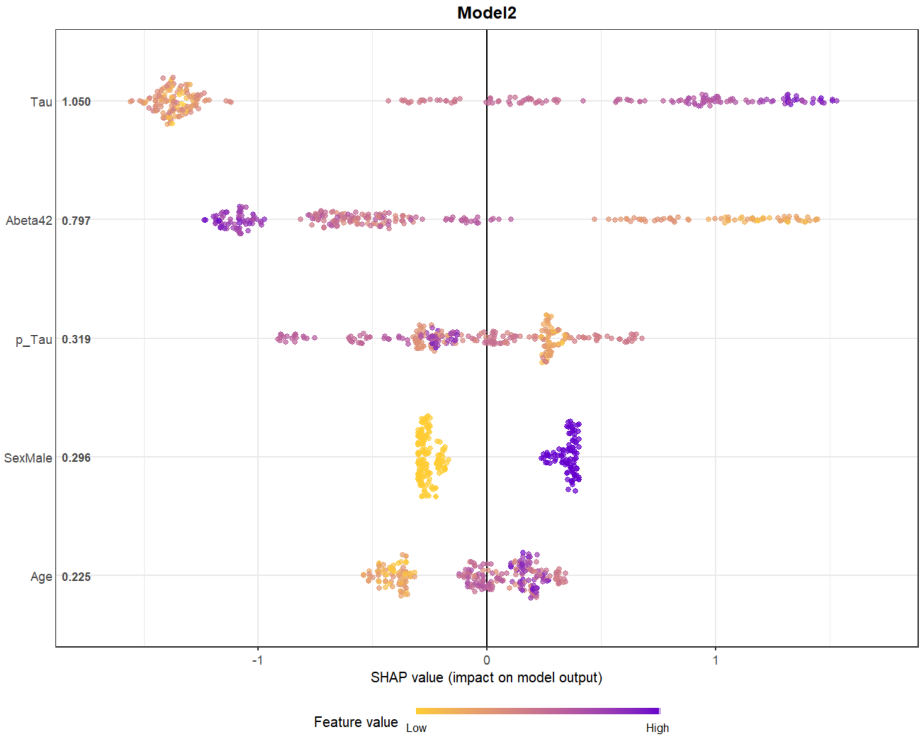

Figure S4. SHAP summary plot for Model 2

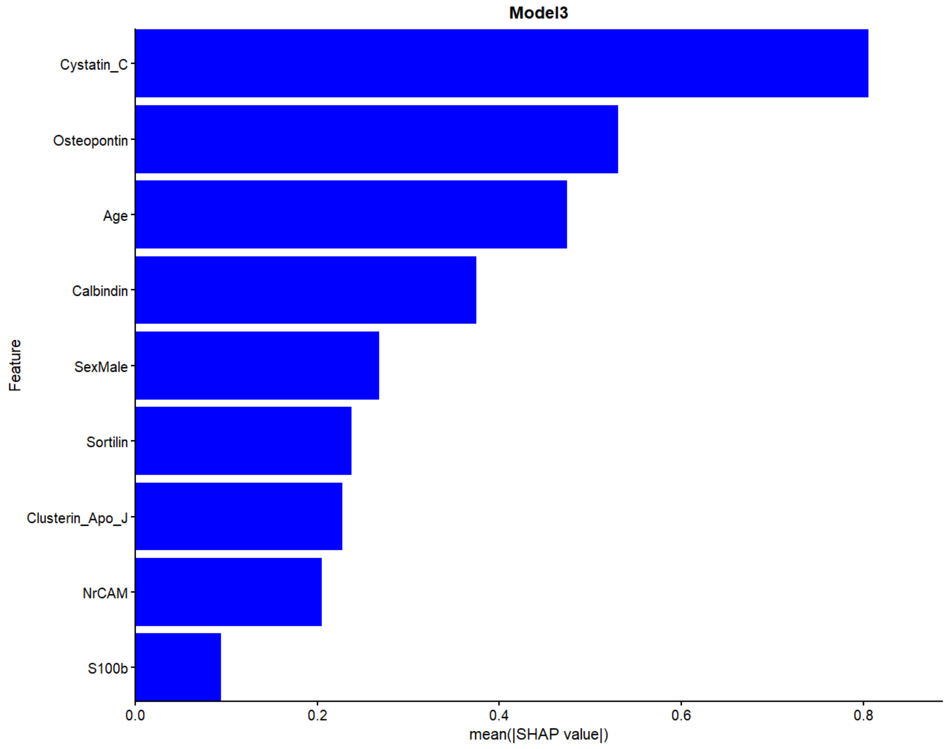

,

Figure S5. Global SHAP feature importance for model 3

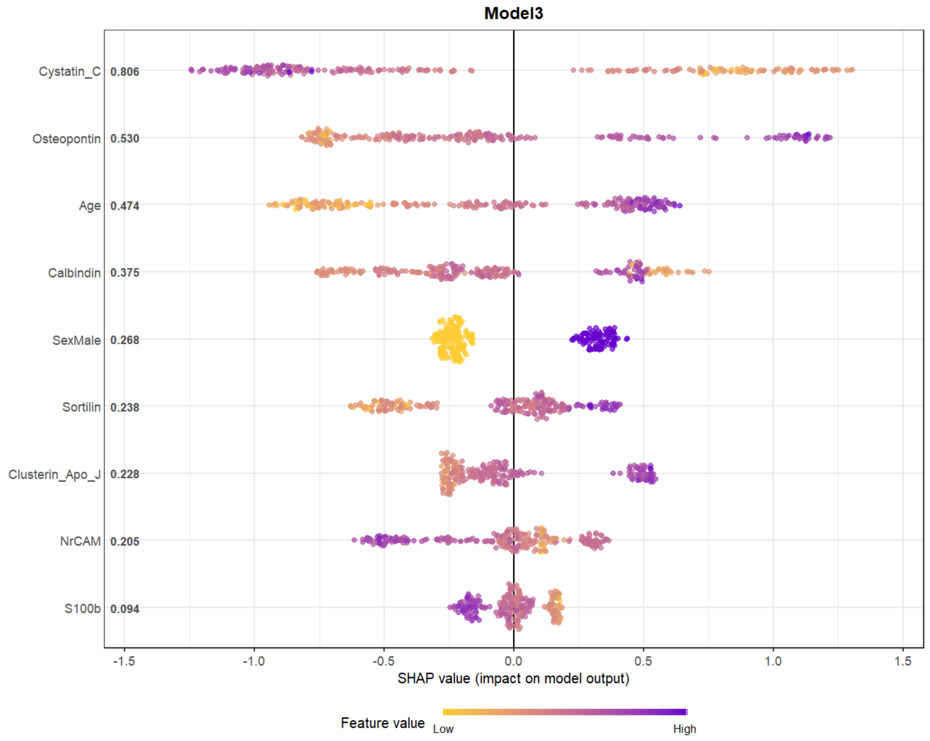

Figure S6. SHAP summary plot for Model 3

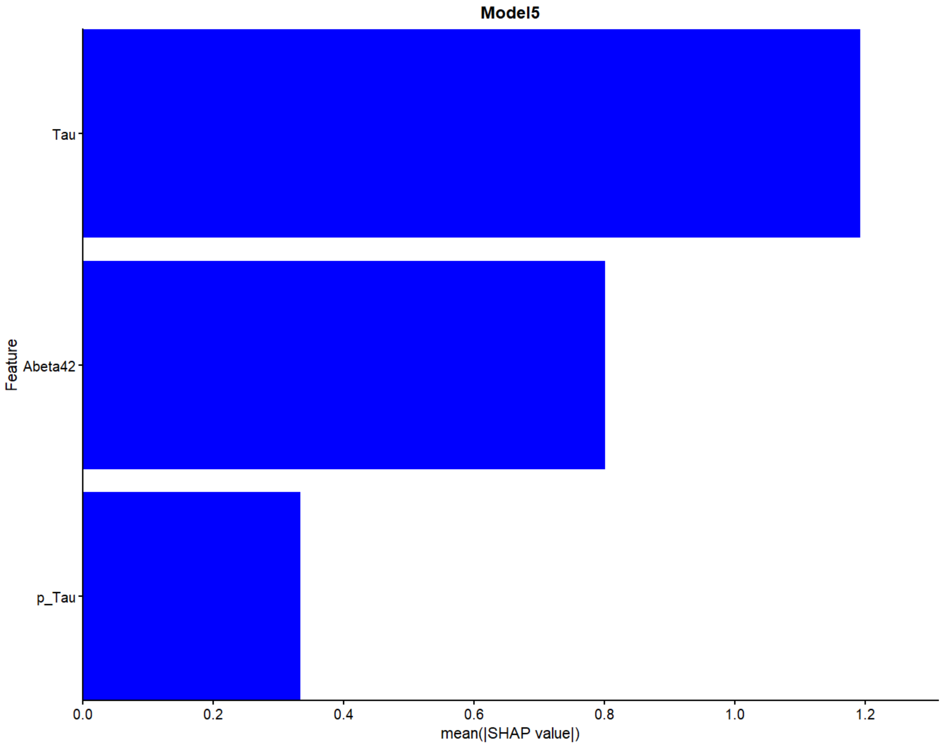

Figure S7. Global SHAP feature importance for model 5

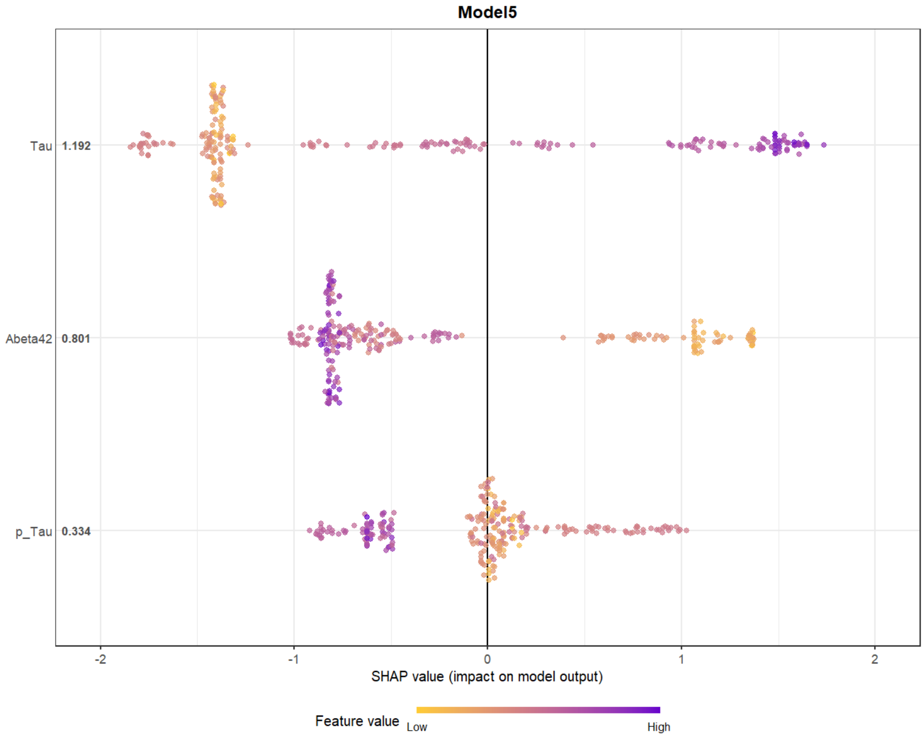

Figure S8. SHAP summary plot for Model 5

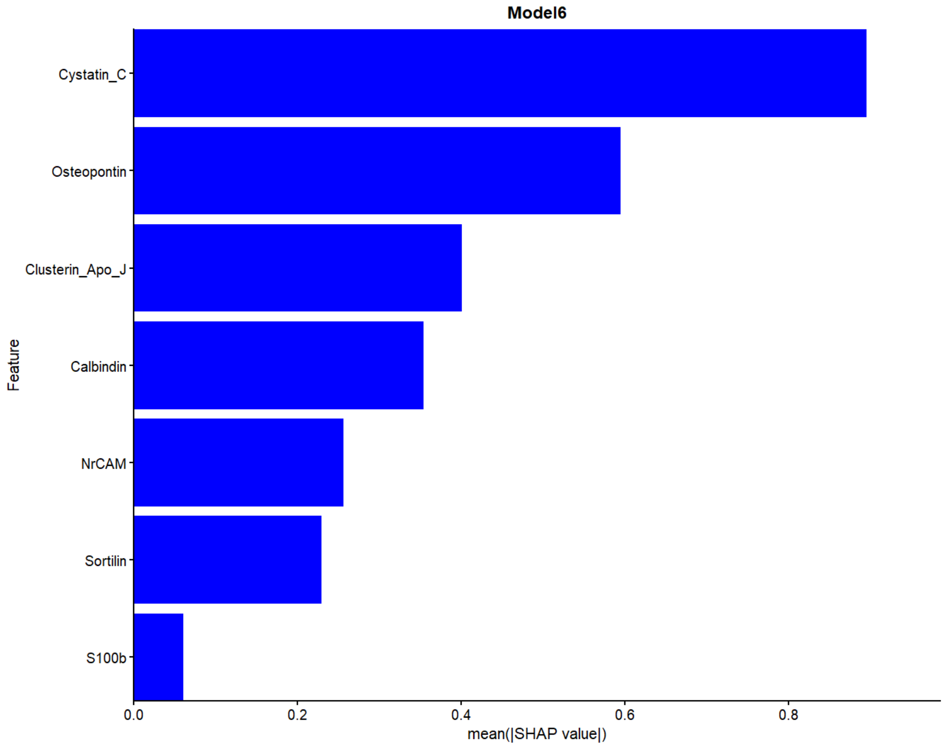

Figure S9. Global SHAP feature importance for model 6

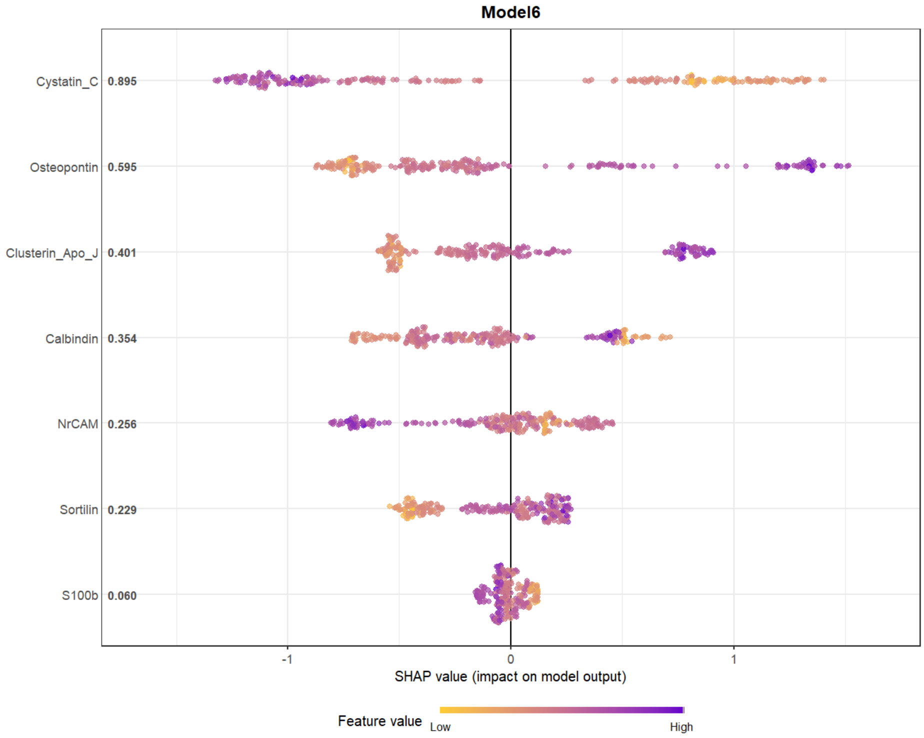

Figure S10. SHAP summary plot for Model 6
